# Context-specific adaptation of an eHealth-facilitated, integrated care model and tailoring its implementation strategies—A mixed-methods study as a part of the SMILe implementation science project

**DOI:** 10.3389/frhs.2022.977564

**Published:** 2023-02-17

**Authors:** Sabine Valenta, Janette Ribaut, Lynn Leppla, Juliane Mielke, Alexandra Teynor, Katharina Koehly, Sabine Gerull, Florian Grossmann, Verena Witzig-Brändli, Sabina De Geest, on behalf of the SMILe study team

**Affiliations:** ^1^ Nursing Science, Department of Public Health, University of Basel, Basel, Switzerland; ^2^ Department of Hematology, University Hospital Basel, Basel, Switzerland; ^3^ Department of Medicine I, Faculty of Medicine, Medical Center University of Freiburg, Freiburg im Breisgau, Germany; ^4^ Faculty of Computer Science, University of Applied Sciences Augsburg, Augsburg, Germany; ^5^ Department of Acute Medicine, Internal Medicine, University Hospital Basel, Basel, Switzerland; ^6^ Department of Hematology, Cantonal Hospital Aarau, Aarau, Switzerland; ^7^ Department of Acute Medicine, University Hospital Basel, Basel, Switzerland; ^8^ Clinic for Medical Oncology and Hematology, University Hospital Zurich, Zurich, Switzerland; ^9^ Department of Primary Care and Public Health, Academic Centre for Nursing and Midwifery, KU Leuven, Leuven, Belgium

**Keywords:** advanced practice nursing, allogeneic stem cell transplantation, adaptation, eHealth, implementation science, integrated care, mixed-methods research design, stakeholder participation

## Abstract

**Background:**

Contextually adapting complex interventions and tailoring their implementation strategies is key to a successful and sustainable implementation. While reporting guidelines for adaptations and tailoring exist, less is known about *how to conduct* context-specific adaptations of complex health care interventions.

**Aims:**

To describe in methodological terms how the merging of contextual analysis results (step 1) with stakeholder involvement, and considering overarching regulations (step 2) informed our adaptation of an Integrated Care Model (ICM) for SteM cell transplantatIon faciLitated by *e*Health (SMIL*e*) and the tailoring of its implementation strategies (step 3).

**Methods:**

*Step 1:* We used a mixed-methods design at University Hospital Basel, guided by the Basel Approach for coNtextual ANAlysis (BANANA). *Step 2:* Adaptations of the SMILe-ICM and tailoring of implementation strategies were discussed with an interdisciplinary team (*n* = 28) by considering setting specific and higher-level regulatory scenarios. Usability tests were conducted with patients (*n* = 5) and clinicians (*n* = 4). *Step 3:* Adaptations were conducted by merging our results from steps 1 and 2 using the Framework for Reporting Adaptations and Modifications–Enhanced (FRAME). We tailored implementation strategies according to the Expert Recommendations for Implementing Change (ERIC) compilation.

**Results:**

*Step 1:* Current clinical practice was mostly acute-care-driven. Patients and clinicians valued eHealth-facilitated ICMs to support trustful patient-clinician relationships and the fitting of eHealth components to context-specific needs. *Step 2*: Based on information from project group meetings, adaptations were necessary on the organizational level (e.g., delivery of self-management information). Regulations informed the tailoring of SMILe-ICM`s visit timepoints and content; data protection management was adapted following Swiss regulations; and steering group meetings supported infrastructure access. The usability tests informed further adaptation of technology components. *Step 3:* Following FRAME and ERIC, SMILe-ICM and its implementation strategies were contextually adapted and tailored to setting-specific needs.

**Discussion:**

This study provides a context-driven methodological approach on how to conduct intervention adaptation including the tailoring of its implementation strategies. The revealed meso-, and macro-level differences of the contextual analysis suggest a more targeted approach to enable an in-depth adaptation process. A theory-guided adaptation phase is an important first step and should be sufficiently incorporated and budgeted in implementation science projects.

## 1. Introduction

In recent decades, there has been a growing interest in adapting health care interventions. As implementing adapted interventions is often more efficient than developing new ones for each setting, this saves human, time and financial resources (1, 2). While the current concept of adaption follows one introduced by Rogers in 1995 (3), it is defined diversely in the literature (4). However, all agree that adaption processes are conducted to match the needs of the target population and to improve an intervention's fit, acceptability and effectiveness in the target context (4–7). Based on these adaptations' targets, e.g., content, method of delivery, the surrounding context (including congruity with the target population's culture), they can effectively redefine an intervention (8–10). Adaptations can include deletions, additions, or modifications (5) and can occur proactively (planned) or reactively (11).

Within a systematic review of adaptations of evidence-based public health interventions, Escoffery et al. (5) identified 42 distinct program adaptations. Among the most common reasons for adaptation were cultural changes (64.3%), followed by new target populations (59%) or settings (57%). Few interventions were adapted to improve their feasibility or acceptability; and only 36% of the adaption studies applied existing frameworks to guide their adaption processes. In a more recent scoping review of current adaption practices (8), 84% of identified studies focused on micro- (i.e., individual-) level intervention adaptions; and the majority (73%) did not report using guidelines or frameworks. Numerous frameworks to provide general guidance for planning and evaluating adaptions have recently emerged in the fields of HIV prevention, sexually transmitted infections, pregnancy, and substance abuse prevention (5–7). A 2019 scoping review (6) identified 13 adaptation frameworks with eight common steps: (1) conducting a needs assessment of the target community; (2) searching for and understanding available interventions with similar aims; (3) selecting a specific intervention; (4) deciding which parts require adaptions; (5) making the appropriate adaptions; (6) testing the adapted intervention; (7) implementing the adapted intervention; and (8) evaluating the adapted intervention. However, as the majority of published frameworks lack a theoretical basis and a multilevel contextual focus regarding the adaptation process, their guidance includes important gaps (5–8).

While context is defined as “a set of characteristics and circumstances that consist of active and unique factors and interacts, influences, modifies and facilitates or constrains the intervention and its implementation” (12), most studies focused on adaptation as a stand-alone process, ignoring the interactions between context, implementation, intervention design, and the adaption process itself (7, 8). Complexity arises not only from the characteristics of the chosen intervention (e.g., the number of intervention components and the interactions between them) and of the target context, but also from the interaction between the two (13–16). This is especially true when complex health care interventions are facilitated *via* the “use of information and communication technology for health,” (17) i.e., electronic Health (eHealth) technology (18, 19). Evidence indicates that 44%–67% of patients discontinue their use of offered eHealth tools due to mismatches between the technology and their context, particularly their needs (20–22); and only 0.01% of all available eHealth applications make it into common use (23), with, however, a rising trend within the COVID-19 pandemic (24). More recently published frameworks, such as the revised Medical Research Council (MRC) framework (13) or guidelines including the recently published ADAPT guidance (25) consider context a core element, upon which all four defined intervention research phases (i.e., development or adaptation of an identified intervention, feasibility, evaluation, and implementation of the intervention) depend. Within the updated MRC framework, considering that, because of reciprocal interactions, a complex intervention's effects are often highly dependent on the surrounding context, Skivington et al. (13) describe context as a core component underlying all phases of intervention research (i.e., development or adaptation of an intervention, its feasibility, evaluation, and implementation). As both effectiveness and implementation often depend heavily on context, the ADAPT guidance also describes context as a key component (25) that demands consideration in all steps of an adaptation process. However, while there is growing evidence on conceptual guidance and frameworks for adaptation processes, there is still an existing lack of empirical insights what methodological approach might work to operationalize such guidance (8). And even if guidelines exist on how to report adaptions and modifications, e.g., the Framework for Reporting Adaptations and Modifications-Expanded (FRAME) (9), few, if any have yet specified *how* to conduct the adaptation process and *how* to methodologically merge context-specific information into the adaptation and implementation processes (13).

Implementation science provides a specific methodology to explore aspects of the context as the first step towards implementation (26). This contextual analysis allows the interventionists to map information relevant to later steps (27–30) and to select or the most effective implementation strategies, which offer proven pathways to support successful adoption, implementation, sustainability and scaling up of interventions, programmes or practices in clinical practice (30). Further, it involves specific methodological considerations, e.g., stakeholder involvement —an interactive relationship-building process between researchers and stakeholders. This is intended to facilitate a shared understanding and informed decision-making (31–33). Stakeholder involvement in implementation research is currently gaining increasing attention, as it provides a foundation to upon which both to build the intervention's acceptability and to ensure its sustainability in the target context (8, 34). Still, even while the importance of context has been emphasized and methodology for its analysis has emerged (7), to date, the understanding of complex interventions’ adaptations is understudied in implementation science (6). More detail and guidance are needed on *how* to conduct contextual adaptations of health care interventions and *how to* tailor their implementation strategies (7).

This study aims to fill this gap by reporting on the methods used to adapt an eHealth-facilitated Integrated Care Model (ICM) and to tailor its implementation strategies to the targeted setting based on a case example (35). This will involve combining a contextual analysis with an in-depth adaptation process. More specifically, we applied a three-step approach with the following specific aims for each:

Aim Step 1: to conduct a contextual analysis focusing on (a) current, context-specific practice patterns and patients' needs; (b) patients’ and clinicians' technology openness; and (c) patients' and clinicians' views regarding the challenges, benefits and requirements for implementing an eHealth-facilitated ICM in their setting.
Aim Step 2: to inform the adaptation process by involving key stakeholders and end-users, and by consulting standard operating procedures (SOPs), overarching Swiss and medical device regulations.
Aims Step 3: to merge the results of aim 1 (contextual analysis) and aim 2 (project group meeting results) toinform the final adaptation of the eHealth-facilitated ICM following the Framework for Reporting Adaptations and Modifications–Enhanced (FRAME) (9); and to tailor our implementation strategies based on the Expert Recommendations for Implementing Change (ERIC) compilation (36).

## 2. Materials and methods

### 2.1. Overall design of this study and description of case example

**Figure 1:**
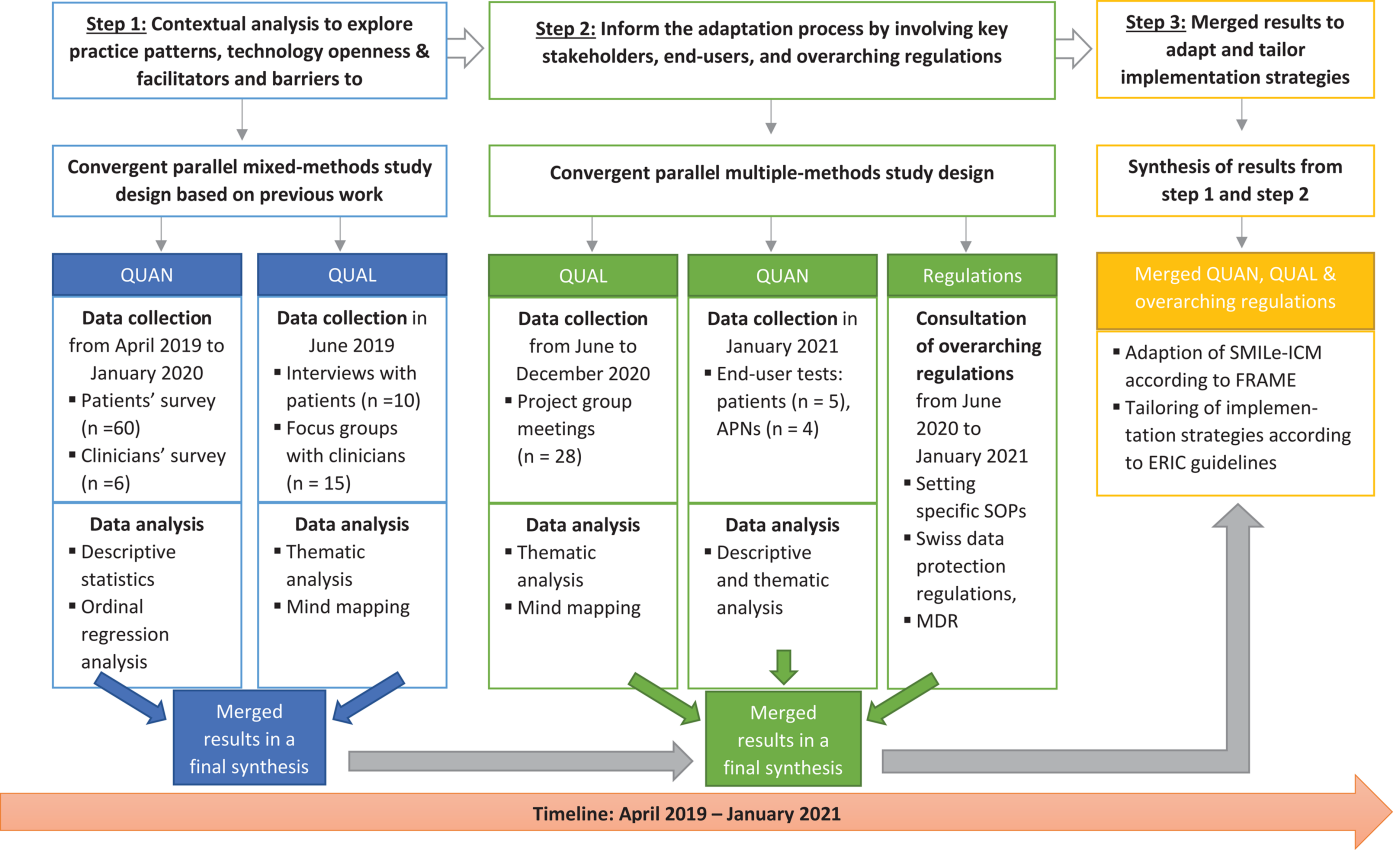
Overview of the study design, data collection timepoints and analysis for steps 1 and 2 to inform the adaptation and tailoring of implementation strategies (step 3). ERIC, Expert Recommendations for Implementing Change; FRAME, Framework for Reporting Adaptations and Modifications–Expanded; MDR, Medical Device Regulation; QUAN, quantitative data; QUAL, qualitative data; SOPs, standard operating procedures.

This multi-level mixed-methods study (37, 38) combined quantitative and qualitative research methods in an equal-status concurrent approach to gain knowledge about the context-specific adaptation of a complex eHealth-facilitated integrated care model. Figure 1 provides an overview of the applied study designs, data collection content and timing, and the analyses for each step. To maximize our understanding of a context-driven adaptation process, we followed a three-step approach. We analyzed first quantitative, then qualitative data, then examined the two merged within a multi-level approach (35).

A nested case study approach was used to generate an in-depth, multi-faceted understanding of a complex issue—intervention adaptation—in a real-life context (39, 40). This approach allowed us to use a case example from an international multicenter implementation science project to develop (41–44), adapt (Phase A), implement and evaluate (Phase B) (45) an Integrated Care Model (ICM) in allogeneic SteM cell transplantatIon faciLitated by *e*Health (SMIL*e*-ICM). Based on an in-depth contextual analysis (41), theory (12, 36, 46, 47) and stakeholder as well as end-user input (42, 43), our study group developed SMILe–ICM for our first participating center in Germany [described in detail elsewhere (41–44)]. For use in the Swiss target setting, this version required various adaptations, including the tailoring of its implementation strategies. In brief, as originally developed, the SMILe-ICM is based on the five building blocks of the eHealth enhanced Chronic Care Model (eCCM) (46). Table 1 describes the SMILe-ICM's original core components, its delivery methods and its delivery timepoints.

**Table 1:** Overview of core components, content, delivery methods and timepoints of the original SMILe-ICM.

**Four core modules**
Monitoring & follow-up of vital signs, symptoms and health behaviour (42, 43)
→ targets alloSCT patients’ insecurities regarding recognizing and reacting to new symptoms; monitors 17 items
Infection prevention (42, 43)
→ targets patients’ challenges regarding infection prevention measures by including (1) adequate hand hygiene; (2) airborne pathogen-related risk reduction; and (3) safe food handling, preparation, and consumption.
Medication adherence (44)
→ targets patients’ immunosuppressant intake (41–43)
Physical activity (42, 43)
→ targets patients’ physical activity alongside their energy levels
**Two delivery modes and detailed intervention description** (42, 43)
(1) SMILe-ICM consists of a technology component, i.e., a mobile app for patients (SMILeApp, initially Android only) and a monitoring interface for care professionals (SMILeCare). In the initial German version (43), patients could insert 17 relevant parameters (i.e., vital signs and symptoms to be checked daily) to the SMILeApp. All data entered to the SMILeApp are transferred to the alloSCT center. With each patient's approval, their input can be overseen by APNs *via* the SMILeCare monitoring interface. This data transfer allows the APNs to monitor, identify and act upon critical values, symptom-related issues or trends based on pre-established cut-offs and risk-adjusted care protocols. Care protocols also specify when other members of the alloSCT team (e.g., responsible physicians, nurses) will be involved. Patients can also read up on important symptoms in the SMILeApp lexicon and receive a step counter to assess daily physical activity. (2) SMILe-ICM is delivered *via* a human part, i.e., APNs. In the original German version, the APNs conducted 12 personal consultations (Visits 1–3 during inpatient stay, visits 4–12 as from outpatient stay) at pre-defined timepoints starting 14 days prior to the patient's alloSCT and extending to one year after. The post-transplant nursing visits are planned in conjunction with the routine outpatient clinic follow-up schedule: While most inpatients attend a 3-week rehabilitation program directly after discharge, outpatient intervention sessions start intensifying 3 weeks after discharge (42, 43)—first weekly, then monthly for stable patients. During these visits, the APN team provides intervention modules on symptom recognition and assessment, infection prevention, physical activity and medication adherence.

Description of SMILe-ICM core components, delivery modes and timepoints/placements of delivery as developed for our first participating center in Freiburg (Germany) (41–44). These will now be adapted for the Swiss context. alloSCT, allogeneic stem cell transplantation; APNs, Advanced Practice Nurses, ICM, Integrated Care Model.

### 2.2. Step 1: Materials and methods for the contextual analysis

As depicted in Figure 1, quantitative and qualitative data for *step 1 (contextual analysis)* were collected from April 2019 to January 2020. The contextual analysis has been approved by the ethics committee [Ethics Committee Northwest and Central Switzerland (EKNZ); BASEC 2019-00307] and is based on previous work by Leppla et al. (41): An in-depth contextual analysis was conducted to inform the development and implementation of the SMILe-ICM to the first participating study's center (Freiburg, Germany) (41).

#### 2.2.1. Theoretical frameworks to guide step 1

***Step 1***. The Basel Approach for Contextual ANAlysis (BANANA) (48) guided the SMILe contextual analysis in our first participating center (41), as well as for this study, which is theoretically based on the Context and Implementation of Complex Interventions (CICI) framework (12). BANANA (48) was developed to provide a step approach to conducting contextual analyses in implementation science projects as follows: choosing a theory, model or framework; using empirical evidence; involving stakeholders; designing a study specifically for the contextual analysis; and determining the relevance of contextual factors for implementation strategies/outcomes and intervention co-design (48). In accordance with the overarching SMILe project (42, 43), the Swiss contextual analysis and adaptation phase was also theoretically based on the eCCM (46), which supports operationalization of all necessary chronic illness management dimensions.

#### 2.2.2. Setting and sample

The contextual analysis was conducted at the Department of Hematology, University Hospital Basel (USB, Switzerland). From April 2019—January 2020, a convenience sampling procedure to survey allogeneic stem cell transplanted (alloSCT) patients from the USB outpatient clinic had been conducted. Eligible patients were: (1) transplanted and followed up at the USB; (2) aged ≥18 years; (3) between six weeks and three years post-alloSCT; (4) able to read and understand German. Those with cognitive or physical impairment that prevented adequate communication were excluded. Clinicians had to meet the following inclusion criteria: (1) >6 months' employment in the transplant center; (2) ≥50% in direct clinical practice; and (3) familiarity with post-transplant care. For individual interviews with patients, the same eligibility criteria applied. Purposive sampling was used to ensure approximately equal variation regarding age, gender, education, and living situation. For the clinicians' survey and focus group sample, clinicians had to meet the following inclusion criteria: (1) >6 months' employment in the transplant center; (2) ≥50% in direct clinical practice; and (3) familiarity with post-transplant care.

#### 2.2.3. Variables and measurement

##### 2.2.3.1. Surveys

Based on the initial contextual analysis (41), patients, clinicians and the transplant director filled in a questionnaire to assess the alloSCT center's structural characteristics, practice patterns regarding chronic illness management, technology openness and perceived importance of eHealth for healthcare applications. Building on previous work by the BRIGHT study team (49, 50) and the PICASSO-TX study (51, 52), Leppla et al. (41) adjusted the questionnaires to the alloSCT setting. Supplementary Table S1 provides an overview of all assessed variables, assessment tools, and their psychometric characteristics, highlighting variables that were adapted and/or added for this study.

##### 2.2.3.2. Interviews and focus groups

*Individual interviews with patients* took place from May until June 2019 to capture a rich understanding of patients` experiences with follow-up care after their alloSCT, their self-management tasks, and their determinants and preferences regarding eHealth application use in their daily lives. Since the aim was to obtain in-depth individual information and understand the personal experience, group discussions (e.g., focus group discussions) were considered as less appropriate in this case. Therefore, semi-structured interviews were conducted by the second author (JR), who is a specially educated nurse in the field of hematology and trained interviewer. Based on previous work (41) and following an interview guide, which has been developed based on eCCM dimensions (46) and CICI framework (12), open-ended questions were asked. These interviews, which took place during the patients' appointments at the USB, were audio-recorded and transcribed verbatim.

*Focus group interviews with clinicians* were conducted in June 2019 to explore their experience with follow-up care and their view of eHealth applications in clinical practice. Since a focus group with amutual exchange of perceptions and expert knowledge can lead to deeper insights into the needs and thoughts of a target group (53) and can generate additional ideas, we have chosen this qualitative approach instead of conducting individual interviews. The focus groups were moderated by the first author (SV), while the second author (JR) mind-mapped key themes on a flip chart to help memorize previous thoughts and summarize all of the focus groups' input (54, 55). During the focus group interviews, the participants could see the emerging maps and could therefore add or change keywords at the end of the focus group session. Both interviewers are experienced qualitative researcher and were trained in conducting focus group sessions. In accordance with Leppla et al.'s earlier contextual analysis (41), clinicians were asked open-ended, semi-structured questions to explore this study's main areas of interest, i.e., we aimed to explore adaptation and implementation requirements for this specific Swiss setting: Therefore, the developed SMILe-ICM for our first participating center (Freiburg, Germany) was briefly illustrated and explained to the clinicians. Afterwards, we then used the group discussion to illuminate what clinicians believe needs to be adapted or further developed for their clinical setting and what is needed to tailor and implement such an eHealth-facilitated ICM for the setting-specific needs.

#### 2.2.4. Data analysis

Descriptive statistics were computed as appropriate for the measurement levels and data distributions (frequencies, means, standard deviations, ranges, medians, interquartile ranges). Secondly, the correlations between the main variable of interest—patients' willingness to use self-management devices in future—and the independent variables—age, gender and education—were analyzed with the Spearman's rho test (age and education) and the Mann-Whitney test (gender). The significance level was set at *p* < 0.05. Significant correlations were tested using logistic regression with a forward approach. Statistical analysis was performed using R Version 3.6.2 (56). Semi-structured interviews with patients were thematically analyzed following Braun et al.'s (57) six-phase procedure. The ATLAS.ti 8 software package was used for data management (58). To analyze the focus groups with clinicians, a mind-mapping technique (54, 55) was applied. As shown in Figure 1, the contextual analysis's QUAN and QUAL results have been merged and synthesized according to the eCCM dimensions (46).

### 2.3. Step 2 and 3: Material and methods to inform and to conduct the adaptation process

As shown in Figure 1, based on integration of the previously-gathered contextual analysis information, multi-stakeholder input and a user-centered design (UCD) approach (59), intervention adaptation and tailoring of implementation strategies were conducted from March 2020—January 2021.

#### 2.3.1. Theoretical frameworks to guide steps 2 and 3

***Step 2.*** To inform the context-specific adaptation of the technology components, we applied UCD techniques (59) by building upon previous work from the iterative software development process (42). Intervention designers place end users' (e.g., patients/caregivers/clinicians) preferences, needs and feedback at the center of each phase of the design process, with the goal of developing or adapting highly usable and accessible products *via* various research and design techniques (60–62). One way of achieving this goal is usability testing very early in the design process, e.g., using interface mock-ups or—later in the process—live applications.

Combining UCD with agile software development enhances its positive aspects. Agile software development offers an iterative, incremental system of software construction (63, 64). While helping researchers focus strongly on creating high-priority functionality, it also acknowledges the value of stakeholder groups, encouraging regular presentations of current product increments to them. Typically leading to particularly safe, effective products, UCD also ensures that the intended users find those products useful and manageable, thereby enhancing their acceptability (65).

***Step 3.*** To theoretically describe the SMILe-ICM's adaptations and why they were necessary, we followed the Framework for Reporting Adaptations and Modifications–Enhanced (FRAME) (9), which provides a coding structure to document types of intervention modifications. Based on literature review, qualitative interviews and stakeholder involvement, Stirman et al. (9) developed a coding system (66) and added additional considerations such as reason for adaptation (e.g., cultural/religious norms, time constraints, access to resources), goal of the adaptation (e.g., increase reach, improve fit) and whether the adaptation was proactive or reactive. The updated FRAME includes eight key components: (1) when and how in the implementation process the modification was made (i.e., timing); (2) whether the modification was proactively planned or reactively unplanned; (3) who participated in adaptation-related decisions; (4) what is modified (i.e., intervention's content, contextual type of delivery, staff training, implementation strategies); (5) at what level of delivery (e.g., individual or unit level) the modification is made; (6) the type or nature of context or content-level modifications (e.g., adding or skipping elements); (7) the extent to which the modification is fidelity-consistent; and (8) the reasons for the modification, including (a) the modification's intent or goal (e.g., to increase reach or fidelity) and (b) contextual factors that influenced the decision (e.g., socio-political factors such as laws or organizational reasons such as staff shortages) (9).

To finally choose implementation strategies for each phase of the SMILe project and to contextually adapt and tailor them for the USB's routine clinical practice, we followed the Expert Recommendations for Implementing Change (ERIC) taxonomy (30), which defines a set of 73 implementation strategies (67). These can be grouped into nine categories: use evaluative and iterative strategies; provide interactive assistance; adapt and tailor to the target context; develop stakeholder relationships; train and educate stakeholders; support clinicians; engage consumers; utilize financial strategies; change infrastructure. In a first step, determinants to implement SMILe-ICM into the Swiss setting has been identified according to micro-, meso-, and macro-level by the interdisciplinary clinical and scientific steering group meetings and categorized in line with CICI framework (12) [overview about determinants are published elsewhere (45)]. We also followed implementation stages according to Exploration, Preparation, Implementation, Sustainment (EPIS) framework (72) to classify the chosen implementation strategies according to the SMILe project's pre-phase, Phases A (development/adaptation) and Phase B (implement and evaluate) and sustainment.

#### 2.3.2. Setting, sample and materials for the adaptation process

Regular project group meetings with key stakeholders, which were led by the first author (SV), were conducted to adapt the SMILe-ICM and to tailor its implementation strategies to the target setting. Therefore, stakeholders were identified following a Stakeholder Analysis Matrix (68): in clinical and research team discussions and brainstorming rounds, we analyzed which internal team members and which external USB staff would be affected by implementing the two main new components (human and technology) of the SMILe-ICM, as well as when (before or after the inpatient stay) and how (directly or indirectly) they would be affected. For each identified stakeholder, we analyzed their impact/influence (low, middle, high) on our project and identified the necessary resources for their engagement (31). For setting-specific adaptations, in addition to stakeholder involvement, we consulted standard operating procedures (SOPs) of the USB's hematology department (69) and higher-level regulatory scenarios [e.g., Swiss data protection regulations (70), medical device regulations (71)].

#### 2.3.3. Quantitative assessment of users' satisfaction

Based on our previous described agile software development process (42) and structured collaboration between nursing scientists and software specialists, a purposive sample of alloSCT patients (*n* = 4), which is described as a sufficient number of participants for end-user usability tests for technology components (72), was formed in 01/2021. To select the sample, SV and JR screened electronic health records to guarantee that different educational levels, genders, and ages were represented, and that the members would be likely to sign a written informed consent form before participation. The four Advanced Practice Nurses (APNs), who would deliver the intervention in test phase B, were approached and recruited by SV and JR.

### 2.4. Synthesis of findings

Results from project group meetings and end-user tests were merged with setting-specific SOPs and overarching Swiss regulations (see Figure 1); then, all were fitted into a meta-matrix (73). This synthesizes contextual analysis results (step 1) based on the eCCM (46) and the results of step 2 to inform the SMILe-ICM adaptation following FRAME (9) and to tailor its implementation strategies according to ERIC guidelines (36).

## 3. Results

According to our three-step approach, our developed methodology will be applied to the results section and described based on the SMILe case example described above (41–44).

### 3.1. Step 1: Results of the contextual analysis

The merged quantitative and qualitative contextual analysis results are briefly described in the following and synthesized in Table 4 in line with eCCM dimensions (46). Supplementary Tables S2, S3 and Figures S1, S2 provide detailed information.

#### 3.1.1. Sample and structural characteristics of the alloSCT center

##### 3.1.1.1. Sample characteristics

**Figure 2:**
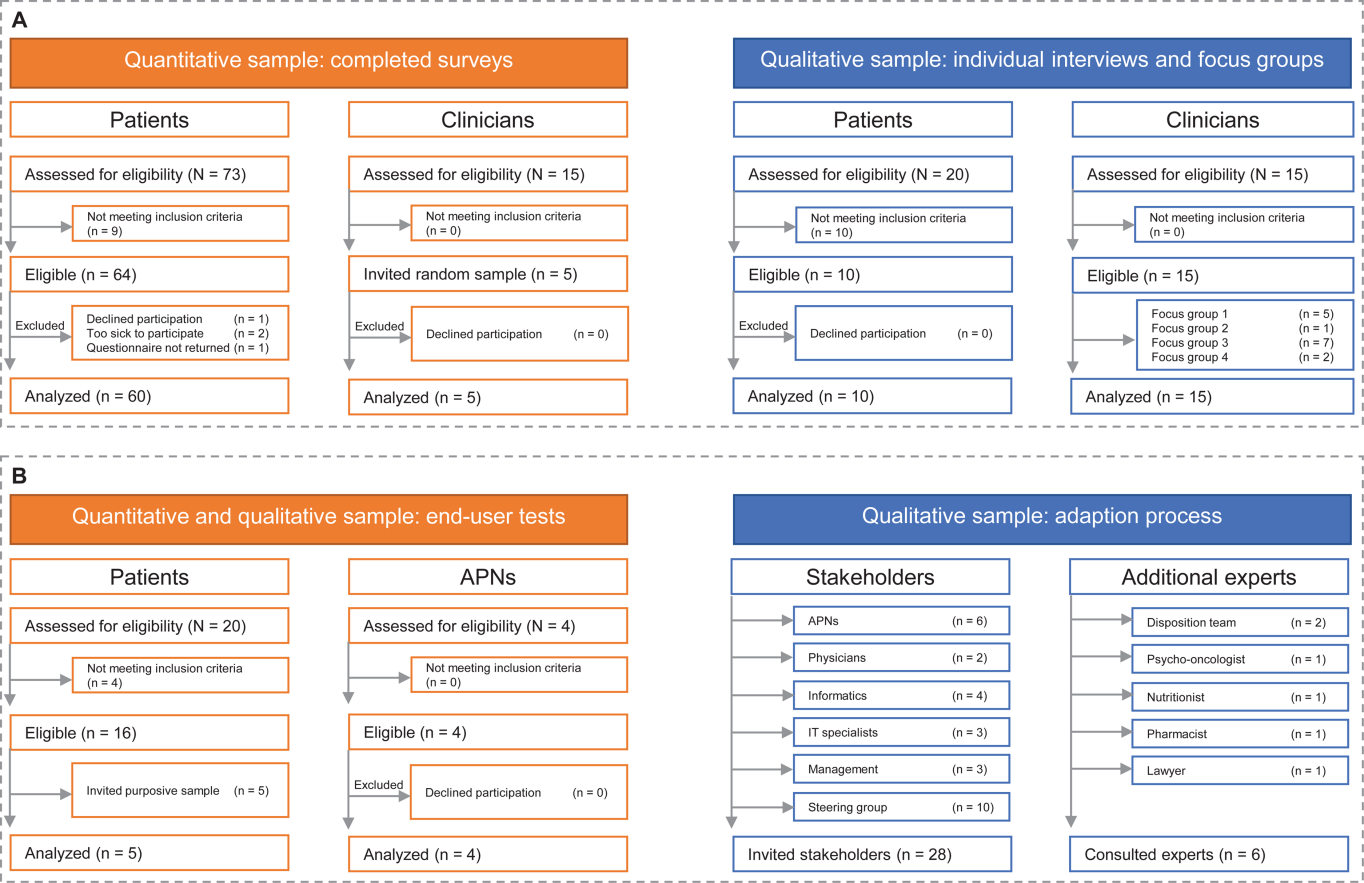
Flow chart of included patients and clinicians. (**A**) Participants within the contextual analysis (step 1). (**B**) Participating patients, stakeholders and additional experts to inform the adaptation process (step 2).

As shown in Figure 2 (flow chart), a convenience sample of 64 eligible patients was invited to participate in the survey (response rate 94%). Of those who accepted, ten were further invited to participate in the individual interviews, which lasted between 40 and 64 min (mean = 48 min); all accepted and participated. For the clinician survey, fifteen HCPs were invited to participate in focus group meetings, all of whom accepted. A random sample of five also agreed to participate in the interviews. Table 2 shows the participating patients' and clinicians' demographic information.

**Table 2:** Demographic characteristics of the participating patients and clinicians.

Patients	Quantitative sample (*n* = 60)		Qualitative sample (*n* = 10)	
Age (in years): mean (SD), range	52 (14.2)	21–74	51 (15)	25–68
Sex *n* (%)				
Male	37	(62)	6	(60)
Time after alloSCT (in months): mean (SD), range	13 (10)	2–36	5 (4)	2–15
Marital Status, *n* (%)				
Married/living with a partner	37	(62)	5	(50)
Single	19	(32)	5	(50)
Divorced/Separated	4	(6)	0	(0)
Educational Level,^a^ *n* (%)				
Tertiary	16	(26)	4	(40)
Secondary II	35	(59)	6	(60)
Secondary I	9	(15)	1	(10)
Employment, *n* (%)				
Unable to work	26	(44)	5	(50)
Retired/student/responsible for household	15	(25)	4	(40)
Part-time	11	(18)	1	(10)
Full-time	8	(13)	0	(0)
Clinicians	Quantitative sample (*n* = 5)		Qualitative sample (*n* = 15)	
Age (in years): mean (SD), range	49 (11)	37–60	51 (10)	37–62
Sex *n* (%)				
Male	1	(20)	3	(20)
Position				
Physician	2	(40)	6	(40)
Nurse	2	(40)	8	(53)
Other	1	(20)	1	(7)
Working experience (in years): median, range	12	6–21	14	5–23

^a^ Education Level: The tertiary level includes the following degrees: University of Applied Science, University. Secondary level II: Apprenticeship, business school/university-entrance diploma.

Secondary level I: no degree, other, secondary school. This allocation of the individual school levels is based on the structure of the Swiss education system (127). alloSCT, allogeneic stem cell transplantation, SD, standard deviation.

##### 3.1.1.2. Structural characteristics of alloSCT center

Located in Northwestern Switzerland, the Department of Hematology of University Hospital Basel (USB) is one of the country's three SCT centers performing not only autologous, but also alloSCTs. The other two are attached to University Hospitals Zurich and Geneva. In Switzerland, about 250 alloSCTs are performed annually (74), of which roughly 100 take place at USB. Several thousand alloSCT patients are currently in follow-up care (75). According to the Swiss Federal Law on Health Insurance, health insurance covers all allowable costs of medical treatment and hospitalization (76). Patients are hospitalized around 10 days prior to alloSCT until a mean of 30 days (±5 days) post-transplantation. After engraftment, patients are discharged once they show stable blood values and health condition. After discharge, they return for follow-up 1–2 times per week for the first 3 months (depending on their health status), then once per week until 6 months post-SCT. Follow-up intervals gradually increase to once yearly. Uniquely for the Swiss setting and as shown in Supplementary Table S2, 42% of patients are additionally followed up in external hematological centers closer to their homes.

#### 3.1.2. Patients' and clinicians' perspectives on current practice patterns

According to synthesized and merged results from patients' and clinicians' surveys, as well as individual patient interviews, three major themes occurred regarding the Basel alloSCT center's practice patterns.

##### 3.1.2.1. Transition to home as most complex phase

Results revealed that follow-up was currently acute-care driven. The most complex phase was seen as the transition to home. While a large majority of patients (93%) generally very satisfied with the provided care, the majority (72%) denied having been contacted by their responsible clinicians after an appointment to ask about their general progress (Supplementary Figure S1A); and 45% indicated that they did not understand the written information they received some or most of the time (Supplementary Figure S1B). While the majority affirmed that they had been advised to adhere to recommendations (Supplementary Figure S1C), 35% did not adhere to checking their cheek temperature on a regular basis (Supplementary Table S2). The mean overall patient-perceived chronic illness management rating was 30.6 (±7.8, range: 11–55). Clinicians' chronic illness management scores (Supplementary Table S2) revealed high variability (mean CIMI-BRIGHT: 2.92 (± 0.58, range: 2.49–3.87). Critical deficits were apparent in 25 items, i.e., <50% positive responses regarding self-management support (10 items), delivery system design (7 items), clinical decision support (5 items) and use of clinical information systems (3 items).

While being generally very satisfied with the care and discharge support they received, patients described the first weeks after discharge from hospital as the most complex, marked by insecurity how to handle self-management recommendations at home. “*Yes, I have about 30 tablets a day. When I was still an inpatient, it wasn't so much; but then it increased and another training would have been helpful.” (female, 42 years)*.

##### 3.1.2.2. Wish for continuous self-management support across the entire patient pathway

A trustful, continuous relationship with the health staff was described by patients as very important. As some experienced frequent changes of assistant doctors, continuity of care was suboptimal. “*Communication and openness, that is the most important thing. But the doctors have changed a bit. (…) And now I'm being cared for by different doctors and that has made it more complicated.”* Further, patients wished they had received more intensive self-management support between the time their alloSCT was approved and the time the actual alloSCT was conducted (which can be months). “*After the patient actually knows that he is going to be transplanted, months can still pass and that might also help to get support there already” (female, 62 years)*.

##### 3.1.2.3. Caregivers' support and burden

Patients reported that, especially in the initial period after discharge, family members provided support with medication management, household chores and transport to and from the hospital. When family members also supported them emotionally, especially when symptoms or side effects of medication occurred, patients also recognized their burden. “*The awareness that things are different now, the family did not realize that right away (…) and all that was of course not easy for them either” (male, 64 years)*.

#### 3.1.3. Patients' and clinicians' technology openness

Contextual analysis results revealed that patients' eHealth openness was high: 81% would be open to try new technologies and 80% would quickly get used to it, while the majority would prefer to receive new applications on their own smartphone (87%) or tablet (46%) (Supplementary Figures S2A,B). As presented in Supplementary Table S2, 50% would be willing to use an App for their medication plan; but 54% would not feel confident entering or updating their medication plans on their own. As shown in Table 3, patients considered the idea of developing new technologies that would allow physicians and nurses to monitor vital signs and symptoms as most important. Higher-educated patients perceived it as more important to develop new technologies that support physical activity (*p* < 0.05); and compared to patients aged over 60 years, those under 60 scored higher on perceived importance of new technologies that provide information (*p* < 0.05, Supplementary Table S3). While all surveyed clinicians (100%, Supplementary Table S2) indicated that written guidelines for care were easily available, electronic medical records were not yet used and no information systems were available to monitor patients at home.

**Table 3:** Patients’ perspective on technology support.

To what extent do you think it's important to develop new technologies that …	Results		
	*N*	Median (25th–75th percentile)	Range
support physical activity?	60	5.5 (3–8)	0–10
measure physical activity?	60	5 (3–7.25)	0–10
give information about healthy eating?	60	7 (5.75–8)	0–10
give information about infection prevention?	60	7.5 (6–8)	0–10
give information about correct hand hygiene?	60	8 (6.75–8)	0–10
regularly request your vital signs?	60	8 (6–9)	0–10
regularly request your physical symptoms?	59	8 (6–9)	0–10
allow doctors and nurses to monitor your vital signs, symptoms and medication intake?	59	9 (7–9)	0–10
remind you of your appointments at the transplantation center?	60	6.5 (3.75–9)	0–10

PICASSO TX Questionnaire; 0–10 Likert scale.

#### 3.1.4. Clinicians' views on the challenges, benefits and requirements for implementing the SMILe-ICM

During the focus groups with clinicians, the current SMILe-ICM version was demonstrated. Afterwards, participants were asked about their perceptions regarding the challenges, benefits and requirements for adapting and implementing the original SMILe-ICM for their setting. Four main topics arose from the focus group interviews:

(1) *Implementation must be based on context-specific requirements.* Clinicians agreed that both human and technology intervention delivery modes should be implemented. However, the entire alloSCT team needs to support this new eHealth-facilitated ICM in daily clinical practice. That will require sufficient resources (staff), as well as APNs who are very well-trained regarding technology and self-management support. Furthermore, the division of tasks should be clearly regulated and open exchanges between participating centers ensured. Clinicians highlighted the advantage of starting the intervention as early as day −14 before alloSCT.
(2) *Human role should be an experienced, trained nurse with competencies in alloSCT care support.* Clinicians stated that the proposed eHealth-facilitated ICM should be provided by APNs, who are trained in self-management support, education and care coordination, and are educated beyond an experienced ward nurse: with extended competencies over the whole patient pathway, they only require support from senior physicians.
(3) *SMILeApp/technology requirements and potential barriers to use.* After having received an overview of the SMILeApp modules' content, all clinicians agreed that, for patients and caregivers, medication management as well as psychosocial support should be included in the SMILe-ICM, which they estimated would be most helpful in the first 3 months after alloSCT. Additionally, barriers to patients' accessibility (e.g., because they are too sick, less educated) to App use should be considered and included in the intervention sessions.
(4) *Costs must be covered to guarantee sustainability.* According to clinicians, such new care models should be fully covered by health insurance to ensure sustainability.

### 3.2. Step 2: results of the in-depth stakeholder- and end-user-involvement to inform the adaptation process

#### 3.2.1. Sample

As shown in Figure 2, an interdisciplinary clinical team from USB, i.e., nurses, physicians, management (*n* = 11), IT specialists and computer scientists (*n* = 7), a clinical and research steering group (*n* = 10), had an in-depth discussion on adapting the SMILe-ICM. Their objective was to coordinate specific processes in two- to four-weekly project group meetings and provide written feedback. Additional clinical experts, i.e., a psycho-oncologist, a nutritionist, a pharmacist, a lawyer originating from the setting and disposition team members were asked to clarify specific questions arising from the project group meetings. Hematology department SOPs (69) and higher-level regulatory scenarios were also consulted [e.g., Swiss data protection regulations (70), medical device regulations (71)] for setting-specific adaptations.

#### 3.2.2. Stakeholder, end-user and overarching regulation involvement to inform differences and needs for adaptation

The combination of project group meetings’ information and setting-specific SOPs informed the adaptation of the SMILe-ICM. Table 4 summarizes the results of the stakeholder involvement and consultation of overarching regulations. This will be described in relation to the adaptation project group meetings' time frames and how micro-, meso- and macro-level information was merged.

**Table 4:** Meta-matrix to inform SMILe-intervention adaptions (according to FRAME) and the tailoring of its implementation strategies (according to ERIC compilation) by integrating synthesized Swiss contextual analysis results as well as findings from project group meetings and overarching Swiss regulations.

Dimensions of the eHealth enhanced Chronic Care Model	Synthesis of findings from step 1: contextual analysis	Synthesis of findings from step 2 to inform adaption and implementation of SMILe-ICM for the USB setting		Nature of adaptation according to FRAME			Implementation strategies according to ERIC guidelines—in bold adapted/added for the Swiss setting
		Source of information	Result	WHAT is modified and at what LEVEL OF DELIVERY	Nature and description of modification	Goal	
**Organizational level**	• Gaps in chronic illness management as no interdisciplinarity • Low- to mid-level chronic illness management	Project group meetings with clinicians and comparison with USB SOPs (69)	In contrast to the first participating center [for which SMILe-ICM was originally developed (41–44)], shorter inpatient stays: Patients at USB hospitalized as from day Tx -10.	– Contextual adaptation—*Timepoint of delivery*	– *Condensing:* Visit 1 starts from day Tx minus10; Session 2 limited to one visit due to short timeframe within inpatient setting	– Improve fit with existing practice patterns (care processes) at USB; Improve feasibility – Improve effectiveness/outcomes	**Pre-Phase** – Access new funding – Develop academic/clinical/technical partnerships – **Inform local opinion leaders** **Phase A** – Identify early adopters – **Visit other sites (e.g., first participating center Freiburg, Germany)** – Conduct local needs assessment and consensus discussion – **Adapt and tailor to Swiss context** – **Adapt educational material** – Organize clinical implementation teams
**Self-manage-ment support**	• Follow up current acute care driven care model (with lack of structured and continued self-management support in follow-up care) • Patients and clinicians wish to add electronic monitoring module for psychosocial and medication management • An eHealth-facilitated ICM is perceived as supportive to help patients overcome their insecurities in recognizing and judging new symptoms, especially after discharge to home	Contextual analysis, project group meetings with clinicians combined with information contained gathered from USB SOPs (69).	In contrast to the first participating center (41–44), no post-discharge rehabilitation program, but direct follow-up appointments after discharge in outpatient setting.	– Contextual adaptation—*Timepoint of delivery*	– *Spreading:* breaking up first intervention session after discharge: intervention session 4 (immediately after discharge) was intensified (to cover visits 4a and 4b) due to regular follow-up visits directly after discharge at USB	– Improve fit with existing practice patterns (care processes) at USB – Improve feasibility – Improve effectiveness outcomes	
		Project group meetings with clinicians combined with information contained gathered from SOPs and expert consultations (i.e., dietician, psycho-oncologist)	In contrast to the first participating center (41–44) and in comparison with USB-SOPs (69): patients receive pre-discharge nutrition and medication management counselling from dietician/pharmacist.	– Contextual adaptions: *Dosing of intervention*	– *Tailoring/condensing:* Intervention session content on SMILe-core “medication management” and ”infection prevention” modules tailored to existing discharge planning at USB: shortening elements that are already delivered by standard care; if required, detailed version given in pre-discharge visit (3rd visit).	– Improve fit with existing practice patterns (care processes) at USB – Improve feasibility – Improve effectiveness outcomes – Address cultural factors	
		Consultation with clinicians/experts: Psycho-oncologist, Senior Physician and dietician	Revealed information to adapt lexicon text and contact details in SMILeApp according to USB SOPs (69)	– Contextual adaptions: *Content adaption*	– *Tailoring:* Refine lexicon entries to Swiss context according to SOPs by keeping core information / elements as original ones, replacing contact details in App that apply to USB-setting	– Improve fit with end-user needs and preferences	
		Usability tests and project group meetings with software developers and usability experts (Augsburg Team)	As in line with first participating center (41–44) and based on USB user test results: need to insert visualization of inserted values for patients’ SMILeApp	– Content: *Adding elements*	– *Integrating:* Technological functionality added = The evolution of entered values (vital signs, symptoms) can now also be viewed by patients on their own devices (mobile phones or tablets).	– Improve fit with end user needs and preferences – Improve feasibility – Improve effectiveness/ outcomes – Address cultural factors	
		Usability tests and project group meetings with software developers/usability experts and nursing scientists/APNs	Similar to first participating center (41–44) and based on contextual analysis and user tests results at USB: adding electronic monitoring module to assess adherence to immunosuppressants and other medication	– Content: *Adding technology functionality*	– *Integrating:* Technological functionality added (electronic monitoring) = patients are additionally asked whether they have taken their immunosuppressive medication and all others as prescribed (yes/no/skip). Patients receive additional electronic monitoring system (128)	– Improve effectiveness/ outcomes – Address cultural factors	
		Usability tests and project group meetings with software developers/usability experts and nursing scientists/APNs	In contrast to the first participating center (41–44) and based on user tests: Revealed information for changing the salutation in the SMILeApp	– Content: *Tailoring technology components to patients’ preferences*	– *Tailoring: Changed* from informal greeting (“Du”) to formal greeting (“Sie”) in SMILeApp	– Address cultural factors – Increase reach or engagement	
**Delivery System Design**	• eHealth-facilitated ICM has been perceived as valuable by patients and clinicians for their setting to improve care and symptom management provided by specialized trained APNs • APNs must be embedded in alloSCT team and ongoing exchange/clear division of work is needed	Project group meetings with IT team, software developers/usability experts and nursing scientists/APNs	In contrast to the first participating center (41–44): IT team members highlighted the need for technical adaptions, i.e., need for an iOS-version instead of providing tablets to patients, who do not have compatible cell phones.	– Content: *Adding technological functionality*	– *Integrating:* Technological functionality added = App is now also compatible with iOS devices.	– Improve effectiveness outcomes – Address cultural factors – Improve fit with recipients – Improve feasibility	
**Clinical Decision Support System**							
**Clinical Information System&eHealth education**	• Patients’ high openness and willingness to use new applications on own smartphone or tablet • Technology needs to be connected to an APN, which is trained in providing eHealth, facilitated self-management support. • Supervision is needed (human and technology)	Swiss data protection regulations (68), and consultation with IT team and USB lawyers.	IT team members and USB lawyers highlighted that the data protection concept formulated for SMILe technology would have to be reformulated and adapted to the “Basel-Stadt Information and Data Protection Law” (78).	– Contextual adaptions: Content adaption	– *Tailoring:* Adaptation of the data protection concept for SMILe Technology to the Swiss setting on the basis of the law on information and data protection.	– Address cultural factors	**Phase A:** – Revise professional roles – Create new clinical teams – Conduct educational meetings **Phase B and sustainment:** – **Ongoing consensus discussion and information of local opinion leaders** – **Provide clinical supervision** – **Provide local technical assistance** – **Remind clinicians** – **Promote spread of clinical innovation**
		Project group meetings with data protection officer, software developers/usability experts and nursing scientists/APNs and consultation with higher-level regulatory scenarios: Swiss data protection regulations (70), medical device regulations (71)	Similar with first participating center (41–44) and according to IT team, data protection officer and overarching regulations [Medical Device Regulation (71)], Swiss data protection regulations (70), planned SMILeApp functionality as medical device was not yet possible due to high costs and organizational efforts	– Content: *Skipping elements*	– Put on hold: Programmed feedback loops in the SMILeApp and monitoring interface were not yet implemented.	– Improve feasibility – Reduce cost	

Synthesis of finding according to Swiss contextual analysis (step 1) and integration of step 2 (adaption process) to inform the adaptation of the SMILe-ICM originally developed for the first participating center (Freiburg, Germany) (41–44), to be adapted to the Swiss setting (Basel, Switzerland) following FRAME (9).

Contextually adapted implementation strategies according to ERIC guidelines (36) regarding the SMILe project's phases A [development (41–44) & adaption] and B [implementation & evaluation (45)]. Implementation strategies written in bold type are those added specifically for the Swiss setting (second participating center, Phases A and B) compared to first participating center (41).

APN, Advanced Practice Nurse, ICM, Integrated Care Model, ERIC, Expert Recommendations for Implementing Change, FRAME, Framework for Reporting Adaptations and Modifications-Expanded, IT team, information technology team, SOPs,standard operating procedures, USB, University Hospital Basel (Switzerland).

##### 3.2.2.1. Findings from clinical expert group meetings merged with setting-specific SOPs

From June to December 2020, the identified clinical expert group—six APNs working in the USB hematological department's in- and outpatient settings—met every 4 weeks to work out the adaptation of the intervention materials to the Swiss setting. Within these meetings, the team compared all written materials for each SMILe intervention session (visit 1–12) with existing setting-specific SOPs (69).

For this purpose, 16 SOPs (e.g., post-alloSCT nutritional, infection prevention recommendations) were consulted. Based on the project groups' meeting exchanges, merged with written feedback from the APNs and considering setting-specific SOPs, SMILe-ICM was adapted and tailored to the Swiss setting as shown in Table 4. In contrast to the first participating center, structured pre-discharge information packages on medication management and dietary recommendations were already included in usual-care discharge planning at the USB. In addition, intervention materials had to be adapted (e.g., the recommendation on wearing an FFP3 mask vs. FFP2 mask) based on SOPs and additional consultation with clinical experts (i.e., dietician, pharmacists).

By comparing the developed intervention sessions and with USB's clinical expert knowledge and SOPs, it also became apparent that, compared to the first center, the wealth of general information on the alloSCT process (e.g., details of the transplantation procedure, possible side effects of chemotherapy) within visit 1 is not possible due to the differences between the two hospitals' usual alloSCT clinical care processes: at the first center, visit 1 takes place as early as d-14 pre-alloSCT. This is not possible in the USB setting, where hospitalization only starts 10 days prior to alloSCT, with numerous examinations taking place on the first two inpatient days. At USB, then, visit 1 is only feasible from d-7, a full week closer to alloSCT.

##### 3.2.2.2. Interdisciplinary meetings with software developers, nursing scientists/clinical nurse specialists and the IT Team

As shown in Figure 2, an interdisciplinary team consisting of three setting-specific IT-specialists, four SMILe-team software engineering developers and two nursing scientists/clinical nurse specialists met every 4–8 weeks (June to October 2021) to discuss adaptations to the SMILe Software components and obtain access to the relevant setting- specific IT infrastructure (i.e., installation of backend components). These meetings revealed a need for additional technical adaptations to fit the Swiss setting, i.e., to compile an iOS-version of the SMILeApp in addition to the original Android version. In terms of implementation, providing tablets to patients who had no Android-enabled cell phones would have been impractical compared to generating an iOS-compatible version. In light of the fact that 47% of the Swiss population use Apple (iOS) smartphones, it would also impact the intervention's sustainability (77).

##### 3.2.2.3. Consultation of overarching regulations

Following Swiss data protection regulations (70), “Swiss Information and Data Protection Law” (78), and in consultation with USB lawyers, the developed data protection concept for the German setting was adapted to the Swiss setting (see Table 4). Although automated feedback algorithms for each parameter (e.g., temperature >38.5: contact the center immediately) had been developed (43), due to Switzerland's strict medical device regulations (enacted in May 2020), these could not be implemented in the Swiss version of the SMILeApp (71). Specifically, the App's feedback loops—both those already developed and those that were planned—would have classified the SMILeApp as a class IIb medical device, which would require extensive and costly certification, additional certification *via* Swissmedic and ongoing quality management (71, 79). The research budget of the project did not allow the investment at that time.

##### 3.2.2.3. Usability tests: end-users' satisfaction

In January 2021, we conducted end-user tests with five patients (mean age 65; 80% male; 100% living in partnerships; education levels ranging from vocational school to master's degree) and four APNs (see Figure 2), who evaluated the SMILe monitoring interface. The tests lasted between 12 and 20 min (mean = 15 min). After completing the tasks, patients and APNs filled in SUS questionnaires. For the APNs, a mean score of 89.5 indicates a very high level of user satisfaction (i.e., scores above 80). Because circumstances of the COVID-19 pandemic made it necessary to conduct our usability tests with patients in a virtual environment, the conditions for the test were sub-optimal, particularly regarding their ability to ask the testers about the questions. As a result, patients' quantitative scores could not be used due to a high rate of missing responses (60%). However, qualitative information from the think-aloud part and subsequent consultations with patients and APNs showed that they perceived both the SMILeApp and the monitoring interface as very intuitive and easy to use. Suggestions for improvement included adding more symptoms (e.g., itching), a medication intake reminder and a change of color, as well as to use the formal forms of German address (Sie/Ihr/etc.) in the SMILeApp.

##### 3.2.2.4. Interdisciplinary clinical and scientific steering group meetings

**Figure 3:**
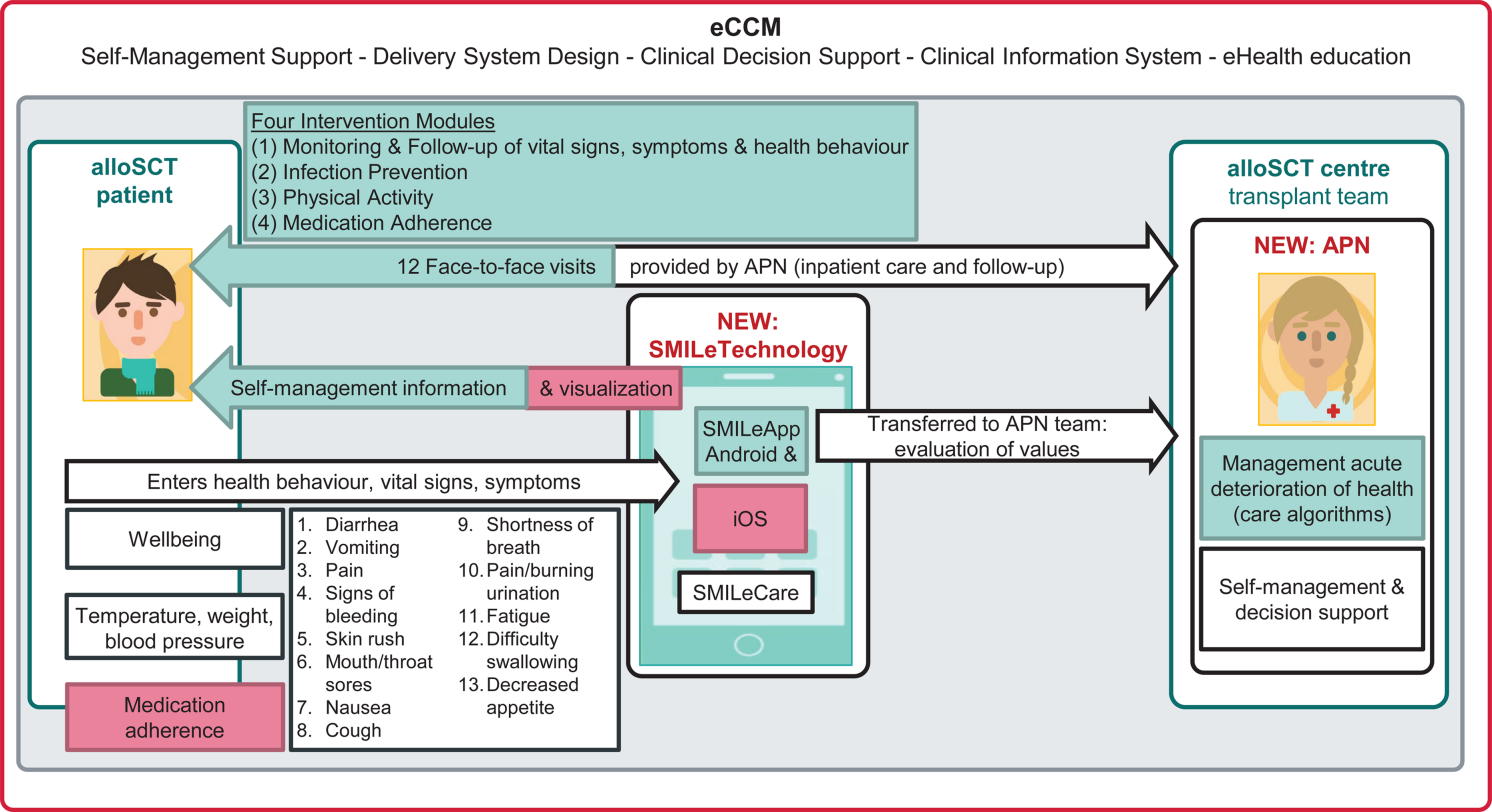
The adapted SMILe-ICM for the Swiss setting. *Note.* Elements of the original SMILe-ICM highlighted in green have been adapted to the Swiss setting. Elements of SMILe-ICM highlighted in red has been added as new functions. alloSCT, allogeneic stem cell transplantation; APN, Advanced Practice Nurse; eCCM,eHealth-enhanced Chronic Care Model.

From June to December 2020, interdisciplinary clinical steering group meetings were held every 4 to 8 weeks. The participants included the clinical expert group (6 APNs), the head nurse of hematology, higher-level nursing management from the Department of Internal Medicine and two senior Hematology physicians (see Figure 3). These meetings had two aims: based on presentations and discussions of the progress and information gathered from project group meetings, to make joint interdisciplinary decisions on modification and setting-specific adjustments; and to support access to infrastructure, such as allocating telephones and workstations for the planned SMILe-APN function. Further, implementation strategies were discussed and tailored to the Swiss setting based on ERIC guidelines.

##### 3.2.2.5. Interdisciplinary scientific steering group meetings

Throughout the phases of the SMILe project, the USB clinical project group leaders (the co-PI SV and JR, who hold joint appointments as APNs in the USB and respectively as a postdoctoral and a PhD student at the university) participated in regular project meetings with their SMILe research team (the PI SDG and other co-PIs, i.e., the developers LL and AT) and scientific team members, every two to four weeks. Within these meetings, project adaptation progress was presented and discussed, joint decisions on adaptations made, and contextually adapted implementation strategies chosen. These meetings also focused on strategic project-related decisions regarding study planning (e.g., third-party funding, preparation of study materials, ethical approval, study registration).

### 3.3. Step 3: merging contextual analysis and project group meeting results to adapt the SMILe-ICM and tailoring of implementation strategies

#### 3.3.1. Adapting SMILe-ICM according to FRAME

The integration of the above-described contextual analysis results, the decisions of the project group meetings, the setting-specific SOPs, the higher-level regulatory scenarios (e.g., Medical Device Regulation) and the usability test results all informed the adaptation of the SMILe-ICM as recommended by FRAME (9). A summary of these processes is available in a Meta-matrix (Table 4). The following paragraphs describe the elements adapted or added to the original SMILe-ICM for the Swiss setting, which are summarized in Figure 3.

##### 3.3.1.1. Timepoint, goal and decision-maker involvement in accordance with FRAME

###### 3.3.1.1.1. Timepoint and planning of adaptations

Following FRAME guidelines, all modifications were proactively planned and executed before implementing the SMILe-ICM in the new setting.

###### 3.3.1.1.2. Involved decision makers and aims regarding SMILe-ICM adaptations

As outlined in Table 3, the interdisciplinary clinical and scientific steering group members, as well as key stakeholders (IT team members, lawyers) made joint decisions as to which SMILe intervention components had to be adapted to fit the USB setting. Core components were kept, but their fit improved regarding the needs of both end user groups (i.e., patients and APNs), existing practice patterns (i.e., care processes) and overall feasibility.

##### 3.3.1.2. Level of FRAME-compliant modifications

###### 3.3.1.2.1. Contextual adaptations for the human component of intervention delivery

As summarized in Table 4, the SMILe-ICM has been contextually adapted due to meso-level (i.e., organizational) and macro-level (i.e., Swiss legal principles) differences between the centers while maintaining its core components. Due to different contact points both pre-alloSCT (d-14 at Freiburg vs. d-10 at USB) and post-discharge, the *timepoints of delivery* had to be adapted to the Swiss setting.

###### 3.3.1.2.2. Contextual adaptations to the technology part

SMILeApp lexicon information and contact details have been adapted based on USB Hematology Department SOPs (69) and in consultation with senior physicians and clinical experts (i.e., psycho-oncologist and dietician). According to Swiss data protection regulations (70), data protection management also required adaptations. Additionally, based on the end-user test results, the salutation pronouns used in the SMILeApp have been changed from informal (“Du”) to formal (“Sie”); and based on the usability test results, in consultation with technology developers, the technology components have been partly adjusted (e.g., the color of the SMILeApp background).

###### 3.3.1.2.3. Context-specific extension for the technology part

Because of the need for SMILeApp to be usable on iPhones [almost half of Swiss mobile telephones (77)], an iOS version was added. Further, based on the results of our contextual analysis and end-user tests, and in line with the first participating center (41, 43), which revealed the wish to add an electronic medication management module as well as an overview of values entered into the app, these technological functionalities have been added to the SMILeApp and its monitoring interface.

###### 3.3.1.2.4. Skipping planned elements

According to Swiss and international regulations (71, 79), certification of the SMILeApp as a medical device was not yet possible for the first participating center (41, 43) and also not yet feasible for the Swiss setting due to limited financial resources. Therefore, neither an already-developed (41, 43) automated feedback system for user-entered values, an automatically updated medication plan for patients, nor color-highlighting of conspicuous values based on predefined cut-offs in the monitoring interface could be realized in this iteration of the app.

#### 3.3.2. Tailoring implementation strategies to the Swiss setting

Based on our synthesis of the key contextual findings and the integration of project group adaptation process results, implementation strategies were tailored to the Swiss setting and classified congruently with the categories used within the overall SMILe project's pre-phase, Phase A (development & adaptation) and Phase B (implementation & evaluation) and sustainment.

As Table 4 indicates, we have chosen seventeen of the 73 ERIC implementation strategies (30). Specifically for Phase A, *an initial local needs assessment,* as well as *creation of partnerships* and *involvement of local opinion leaders* have been recognized as essential (see Table 4 for all chosen implementation strategies). Especially regarding adaptation, it became evident that *visiting other sites, adaptation and tailoring to the Swiss context and the organization of clinical implementation teams* are all crucial to a context-specific implementation. In combination with this context's low level of chronic illness management, the perceived requirements for new clinical roles (i.e., context-specific APN training) postulated *the creation of new clinical teams and conduction of educational meetings*.

Further implementation strategies were formulated to support the project's Phase B [i.e., implementation, evaluation (45)]. In addition to *ongoing consensus discussion and informing of local opinion leaders, provision of clinical supervision, and provision of local technical assistance, these included provision of reminders for clinicians and dissemination of clinical innovation.* Merging contextual analysis results with stakeholder involvement discussion outcomes revealed that ongoing exchanges, supervisions and support are all needed to ensure the SMILe-ICM's implementation and long-term sustainability.

## 4. Discussion

With this mixed-methods study, we aimed to contribute to the understanding *on how to* contextually adapt complex interventions and tailor implementation strategies, what is so far understudied in the field of implementation science (6). Our elaborated, step-by-step methodological approach combines a theory-driven contextual analysis (48) with the in-depth, theoretically framed (9) adaptation process of a complex intervention, explaining how to tailor its implementation strategies based on recommendations from implementation science methodology (30) for any context in real-world settings.

### 4.1. Reflections and implications from our step-wise approach

In recent years, context has become a central concept in adapting health care interventions (80, 81). In our first step, a methodologically grounded contextual analysis following BANANA (48) supported our understanding of current clinical practice patterns, as well as of clinicians' and patients' views on their needs and technology openness. BANANA had already proved useful for the contextual analysis of our first participating center (Freiburg, Germany) (41). It also helped us to conduct a profound evaluation of contextual aspects in the current study (in Basel) (48). While our contextual analysis confirmed the need, wish and openness to implement the first SMILe–ICM (42, 43) into the Swiss setting, its results revealed predominantly similar findings to those of the Freiburg setting (41): current clinical practice was rather acute-care-driven, with a similar PACIC rating [Basel = 30.6, Freiburg = 32.6 (41)] and CIMI BRIGHT scores [Basel = 2.92, Freiburg = 2.74 (41)]. As in the Freiburg setting (41), patients highlighted a wish to have continuous self-management support across the entire patient pathway. This is in line with previous evidence calling for patient-centered, continued care coordination, especially for the complex posttransplant transition phase—the period during which patients are transferred from full in-patient support to full individual responsibility for self-management tasks at home (82–84). Concerning eHealth openness, this study's contextual analysis results were quite similar to those obtained for Freiburg (41), and are congruent with evidence (85–87) supporting cancer patients' openness to eHealth-applications. I.e., patients support eHealth as long as personal contact is maintained. Considering that most existing technology's efficacy depends largely on its link to timely and personal health care provider responses, evidence supports the planned integration of eHealth and human support (18, 19, 85–87). In turn, this may lead to closer patient involvement in shared decision-making processes, as well as to faster communication and therefore to increased overall satisfaction (86, 88–91).

However, within our second step—the adaption phase, i.e., the in-depth exchange with key stakeholders merged with information from the meso- [i.e., setting specific SOPs (69)] and macro-levels [i.e., Medical Device Regulation (71), Swiss data protection regulations (70) and cantonal data protection law (78)]—we discovered considerable differences between the centers' practice patterns and organizational structures. Such variability in practice patterns at alloSCT centers is also described in the literature—although the related information is still limited and based primarily on evidence from the U.S. (92–96). Still, with our first step, i.e., a context-specific needs assessment and the exploration of clinicians' and patients' perspectives regarding current practice patterns in the target setting, we obviously missed a crucial component: information about the institutional procedures and how these differ between settings. Details of these procedures were important to inform the adaptation of our eHealth-facilitated ICM. Based on our results, then, we would suggest that even in the first steps, differences between the contexts should be mapped in terms of meso-level information (i.e., characterization of usual practice patterns) and macro level specifications (i.e., legal requirements). Ideally, detailed information on the selected intervention, its essential components and important delivery modes should be obtained in advance. This could then be combined with an exploration of targeted meso- and macro-level information by involving diverse key stakeholders, e.g., clinical experts, policy stakeholders and potential end-users, as early as possible (25) in focus group or individual interviews. This step could lead into a tightly-defined contextual analysis with targeted research questions chosen to inform the open needs of the adaptation and implementation phases, i.e., to shorten the investigations on the individual patient and clinician level by collecting better-targeted information and focusing more on meso- and macro-level aspects as it is also suggested by the recently published ADAPT guidance (76): Even in their first step, in order to minimize the necessary time and personnel expenditure for a context-specific adaptation, the authors suggest mapping similarities and differences between original and new contexts (76).

In terms of providing a foundation to facilitate the acceptability and sustainability of developed or adapted intervention in new contexts, stakeholder involvement is rapidly gaining acceptance as an indispensable tool (8, 34). Our in-depth stakeholder involvement was also central to our intervention adaptation (step 2): Throughout the adaptation phase, our stakeholder group meetings brought diverse perspectives on how to adapt our eHealth-facilitated ICM. To meet the needs of clinicians, patients, and researchers, every available perspective was necessary.

Indeed, our adaptation process, including its in-depth initial contextual analysis and broad stakeholder involvement, took us twenty-one months to complete. While previous research suggests allowing 6–9 months to adapt an intervention (4, 8, 25, 81, 97), no systematic overview and comparison of the time and effort it takes to adapt complex health care interventions yet exists. Such an overview would allow a clear record of the adaptation process's duration and effort. However, to allow cross-study comparison of these variables, it would be necessary to consider the investment not only of time, but also of personnel (e.g., percentage of staff, number of employees). Such details are even less available in the literature than those regarding time investment. To lower the cost, the literature also discusses “rapid methods” to adapt and optimize an intervention in a fast-changing context (98, 99). To inform quick adaptation and optimization of behavioral interventions in evolving public health contexts, within a short timeframe of 1–2 months, Morton et al. (100) used rapid methods to modify the online “Germ Defence” intervention (101). However, it cannot be assumed that every complex intervention can be rapidly adapted and implemented in every new context: every new context's norms, resources, and delivery structures differ from those of the original (99). Especially regarding the adaptation and implementation of eHealth-facilitated ICMs, evidence suggests that, to understand the complex adaptation processes used in implementation science projects, health information technology adaptation research should apply multilevel and multidimensional evaluation instruments (99). Consistent with our study design, mixed-methods approaches that involve key stakeholders to explore the dynamic relationships between technology and social factors are needed (102, 103).

While stakeholder inclusion is an adaptation process that helps ensure that the approach is appropriate, feasible, and acceptable (104), it is also time-, resource- and effort-intensive (31, 105). For interventions in chaotic, resource-competitive clinical settings—particularly compared to the controlled environment of a research setting—including stakeholders is especially difficult (99, 106): numerous environmental factors (especially limited time and funding) can act as barriers to participation in clinical research projects (107, 108). For example, for many stakeholders, involvement requires skills and competences both to present their own opinions and interests and to argue for or against those of others (31, 105). However, within adaptation guidance papers or studies, reflections on how to deal with differences of opinion are scarce (8). For our approach, a combination of favorable factors—including strong joint leadership engagement, both from the clinical nursing management and from the research infrastructure staff, a well-established academic-practice partnership between the clinical setting and the research institute (109) and the fact that implementers worked partly in clinical settings and partly in academic institutions—have all been identified as strong facilitators for our adaptation and implementation process (45). Moreover, this dynamic combination of people and competencies supported us in realizing a shared vision and commitment among interprofessional stakeholders. And while clinical stakeholders bring in-depth clinical expertise and setting-specific knowledge to the table, academic partners often provide access to funding sources and to expert researchers (110, 111).

However, such resource-intensive multiple-methods study designs with in-depth involvement of key stakeholders commonly suffer from limited funding possibilities (105), which can impede widespread adoption for even the most well-positioned innovations (112–115). Therefore, specific funder commitments must be secured to adequately support such projects—not only to the stage of clinical trials, but all through the adaptation and implementation phases (31, 116) to ensure sustainability (99, 117). In terms of lowering costs and efforts, another emerging opportunity to support complex eHealth adaptation and implementation studies is the application of big routine data sets (99, 118). Automated processing of such data (e.g., hospitals' electronic health records) or socio-economic figures (e.g., national-level statistics on age, education, occupation, income) could quickly distill macro-level information from regularly-updated data on large, diverse populations at low cost (119). As part of our third step, FRAME provided guidance on how to report adaptations and modifications, including several cursory indications of which aspects should be considered in the adaptation process (e.g., who participates in the decision to modify, what should be modified) (9). Consistent with previous studies applying FRAME's (120–122) coding structure, FRAME (9) was particularly useful for the structured, step-by-step tracking of adaptations to our eHealth-facilitated ICM. And from the earliest stages of our adaptation and implementation process, ERIC recommendations (30) supported us both to choose and to describe context-specific implementation strategies. Since this project's launch, a new framework for documenting adaptations to implementation strategies has been released—the 2021 Framework for Reporting Adaptations and Modifications to Evidence-based Implementation Strategies (FRAME-IS) (123). Developed on the basis of the existing FRAME (9), elements of FRAME-IS (123) closely mirror those of the original (9), but with language specifying, for example, that modifications are made to implementation strategies, not to the overall intervention. While the authors suggest using the ERIC compilation (30) within the FRAME-IS, this tool provides guidance not only to choose implementation strategies from ERIC's offerings (30), but also to document and justify modifications to implementation strategies. These steps could be supportive of any further adaptation and implementation science projects. In line with recently published recommendations (124) suggesting to identify multiple barriers that can support a structured approach to choose a wide range of implementation strategies supported also the tailoring of our implementation strategies in addition to the stakeholder involvement and the application of theoretical frameworks for categorization (i.e., EPIS (125) and CICI (12) framework).

### 4.2. Strengths and limitations

This study's most notable strength is its comprehensive multilevel mixed-methods approach (37, 38), which facilitated the gathering, merging and interpretation of context-specific qualitative and quantitative information. Further, our in-depth adaptation process not only informed the theoretically framed adaptations (following FRAME) (9), but also enabled us to tailor implementation strategies to the new context following ERIC compilations (30). This innovative approach bridges important gaps in terms both of clinical innovation for complex, eHealth-facilitated care delivery and of intervention methodology.

Despite such promising elements, this study has certain limitations. First, realities of clinical practice, e.g., changes in personnel and/or leadership and especially the unforeseen crisis of the COVID-19 pandemic can affect the success of any implementation. Therefore, an ongoing analysis of practice patterns and change, which was not systematically planned within our approach, will be needed. However, within our implementation and evaluation phase (45), we have continued to conduct regular stakeholder group meetings with the involved clinical management, the research steering group and the APNs: this belongs to our implementation strategies for Phase B (i.e., ongoing consensus discussions and information from local opinion leaders, provide clinical supervision, provide local technical assistance, remind clinicians, spread clinical innovation).

Second, due to COVID-19 pandemic regulations, we were forced to conduct the usability tests (step 2) in a virtual environment. Due to this adverse condition, the quantitative results have to be considered with caution. Nevertheless, the qualitative information from the think-aloud part and subsequent consultations with patients and APNs gave us insightful and repetitive information on adapting the technological component of the SMILe-ICM.

Limitations of our human and time resources prevented the completion of the digitalization process as originally planned. According to the Medical Device Regulation introduced in May 2020 (71), the SMILeApp could not yet be classified as a Medical Device (126). However, while we are currently evaluating the eHealth-facilitated ICM in our two participating centers (45), we are confident that the methods used will increase the probability of sustainable implementation and acceptance in real-world clinical practice.

### 4.3. Implications for research, practice, and policy

Adapting a health care intervention that integrates eHealth-facilitated components is a complex undertaking. All tailoring must focus specifically on the target context and a local implementation (99). For this purpose, we have developed a step-wise mixed-methods adaptation approach that features strong stakeholder involvement that is in line with the recently-published ADAPT guidance (25),. Oriented towards a comprehensive knowledge of key contextual factors, this approach will help to operationalize and structure adaptation processes, facilitating shared understanding and informed decision-making (31–33). For example, as this study's results indicate that critical differences were predominantly at the macro level rather than at that of patients and clinicians, directing our first step at that level—mapping contextual differences between the two test centers with a focus on macro-level factors (e.g., differences in existing care processes along the patient pathway)—would have saved time and resources.

Additional research is warranted to explore changes in contextual factors using mixed-methods evaluations across multiple data collection periods. For example, in addition to quantitative data monitoring, regular focus group meetings with key stakeholders could identify critical contextual developments—particularly issues arising in the adapted intervention's implementation or execution—at an early stage (102, 103).

In light of practical and policy implications, establishing academic-service partnerships and involving key stakeholders throughout the adaptation process are key implementation strategies for an implementation science adaptation project; therefore, these should be considered and planned from the beginning (8, 34). Convincing policymakers and funders to adequately support in-depth adaptation processes and the contextual analyses that guide them, more studies will need to report transparently on the related processes, their timelines and their long-term value (31, 116). Overcoming barriers to the adaption and adoption of eHealth-facilitated care, particularly the currently prohibitive Medical Device Regulation`s requirements and reimbursement policies (24), will require key changes in policy priorities, beginning with a system-level definition of innovation, a clear overarching mission and efficiently-aligned funding structures (99, 117).

## 5. Conclusion

This study describes our development of step-wise implementation-science-based methodological approach on how to conduct a context-driven, theoretically framed intervention adaptation, including the tailoring of its implementation strategies to the target setting. As this is an example of implementation science methodology, its underlying principles—particularly its end-user focus, its extensive use of stakeholder involvement, and the value it places on contextual knowledge—can be applied directly both to study environments and to real-life settings. Further, it is the product of a multidisciplinary effort led by both clinicians and researchers. To adapt and implement a complex intervention into daily clinical practice, the study project employed the perspectives of clinical health care professionals, IT team members and patients.

Our experience emphasizes that a contextual analysis is essential to understand current needs, practice patterns and openness towards an intervention's implementation. However, in light of our discovery that certain meso- and macro level differences were actually critical to adaptation, we strongly recommend that the contextual analysis include information on both levels (i.e., exploring contextual differences) from the beginning. This can be done by discussing relevant issues with key stakeholders and clinical experts, then using their input to map out targeted meso-and macro-level differences or similarities from the beginning.

In spite of having to correct for this omission, our approach finally supported a smooth implementation (i.e., with high acceptability and adoption) of the adapted eHealth-facilitated ICM into practice, thereby increasing the likelihood of its sustainability. Therefore, we conclude that a well-planned, theory-guided, contextually tailored adaptation phase provides a vital step towards an intervention's successful, sustainable implementation. As this relies strongly on a comprehensive knowledge of the target context, an in-depth contextual analysis should be incorporated and budgeted in every implementation science project.

## Data Availability

The original contributions presented in the study are included in the article/supplementary materials. Further inquiries can be directed to the corresponding author.
