# Supplementary material for: Context-specific adaptation of an eHealth-facilitated, integrated care model and tailoring its implementation strategies—A mixed-methods study as a part of the SMILe implementation science project
Source: Front Health Serv. 2023 Feb 17;2:977564. doi: 10.3389/frhs.2022.977564 (PMC10012712; doi:10.3389/frhs.2022.977564)
Supplement: Supplementary file 1 [file Table1.pdf]

Supplementary Table 1. Overview of assessed variables, assessment tools and their psychometric characteristics

1. Patients' survey

Dimension	Items	Variables	Instrument/measurement<br>Values/scoring/conceptual definition	Psychometrics	References
Demographic characteristics	Items 1-7	Age (year of birth)			(1, 2)
		Gender			
		Marital status			
		**Educational level**			
		Employment status			
		**Disability pension**			
Health behavior	Items 8-40	**Level of physical activity**	BRIGHT patient self-report, adapted to alloSCT Swiss setting; patient self-report; 3 items, advice to physical activity (yes/no); frequency of moderate activity, frequency of sports activity. Sufficiently active: ≥ 3x/week 20 min. of vigorous AND/OR ≥ 5x/week 30 min. of moderate physical activity	Criterion validity<br>K statistic 0.14-0.40<br>moderate inter-rater reliability<br>(κ= 0.53, 95% CI= 0.33-0.72)	(1-4)
		**Currently smoking**	BRIGHT patient self-report, adapted to alloSCT setting;1 item; self-report; 4-point Likert scale (1 = yes to 4 = no, never before)	No information	(1-3, 5)
		**Level of alcohol consumption**	BRIGHT patient self-report, adapted to alloSCT Swiss setting; 4 Items; 'Yes/No' advice by transplant team, drinking alcohol (4-point Likert scale: 1 = yes to 4 = no, never); If yes: Frequency (1 item ranging from 1/day to 3x/per month) and quantity (1 item: 1dl to 1 liter or above)	No information	(1-4)
		**Sun protection measures and adherence to sun protection recommendations**	BRIGHT patient self-report, adapted to alloSCT Swiss setting; 5 items; 'Yes/No' advice by transplant team, sun-exposure (in time) and sun-protection usage (Sun crème and head protection) on 5-point Likert scale ranging from '1= never' to '5= always'	Unidimensional scale, Cronbach's alpha of 0.59	(1-3, 6)
		**Adherence to food recommendations**	BRIGHT patient self-report, adapted to alloSCT Swiss setting; 8 Items, 5-point Likert	No information	**(1-3)**

			scales: never, seldom, sometimes, often, always; often-always considered as yes.		
		**Adherence to infection prevention**	BRIGHT patient self-report, adapted to alloSCT Swiss setting;8 Items, 5-point Likert scales: never, seldom, sometimes, often, always; often-always considered as yes.	No information	**(1-3)**
Practice Patterns: Experiences with follow-up care and team	Items 41 - 63	**Placement of follow-up care appointment**	BRIGHT patient self-report, adapted to alloSCT Swiss setting; 1 Item asking for follow-up care only at the TX center (yes/no); if not, specify which external center.	No information	(1, 2)
		**Adherence to appointment keeping**	BRIGHT patient self-report, adapted to alloSCT Swiss setting; 2 Item asking for adherence at TX center and/or external center, 5-point Likert scale: 1= never missed to 6= last 5 appointments missed.	No information	(1, 2)
		**Time spends with the transplant team during follow-up**	BRIGHT patient self-report, adapted to alloSCT Swiss setting; Time spentt with the transplant team during follow-up, 2 items asking for time spent with physicians and nurses (in minutes) and further HCP (psycho-oncologist, dietician, social worker, other, in minutes)	No information	(1, 2)
		Patients' perspective on Self-Management Support and CIM	**Short version of the Patient Assessment of Chronic Illness Care (PACIC):** 11 items, 5-point Likert scale: 1) Self-Management Support (8 items)<br>2) Delivery System Design and Clinical Decision Support (1 items)<br>3) Most CCM components (2 items)1= none of the time to 5= all of the time.	Unidimensional scale, with a Cronbach's alpha of 0.88	(1, 2, 7)
		Health Literacy	BRIGHT patient self-report; 3 items, 5-point Likert scale: 1= none of the time to 5= all of the time.	Concurrent validity: with the Short Test of Functional Health Literacy in (AUC=.72-.74; with the Rapid Estimate of Adult Literacy in Medicine (AUC=.81-.84)	
Patients' perspective on eHealth:	Items 64-113	Devices types<br>(1 item)	PICASSO TX Questionnaire patient self-report: Nominal variable, with multiple choice answers	No information	(8, 9)

EHealth experience and usage patterns			(mobile phone, smartphone, tablet pc, laptop, pc, smartwatch)		
		Place of use	Ordinal variable, with multiple choice answers (at work, private, others, at work and at home)	No information	
		Duration of ownership	Ordinal variable with single choice answer (< 1 month to > 3 years)	No information	
		Frequency of use	Ordinal variable with single choice answer (never to daily)	No information	
Patients' perspective on eHealth: EHealth openness and overall willingness to use an eHealth self-management device	Items 114-133	Willingness to use Apps to monitor symptoms and behavior after alloSCT	PICASSO TX Questionnaire adapted to Swiss alloSCT patients: 21 items;<br>5-point Likert scale /ordinal (0 = not at all to 5 = conceivable at all)	No information	(8, 9)
	134a.-i	**Patients' willingness to use self-management devices in future as support in physical activities, querying vital signs and symptoms, nutrition, hand disinfection, time scheduling**	9 items: 10-point Likert scale / ordinal (0 = disagree to 10 = agree)	No information	
	135 a.-h.	**Need for new technologies for chronic illness self-management (pedometer, robotic)**	Adapted items from BOPS questionnaire INSPIRE study: Nominal variable with single choice answer (yes, no)	No information	(10)
**2. Clinicians' survey**					
**Dimension**	**Items**	**Variables**	**Instrument/measurement Values/scoring/conceptual definition**	**Psychometrics**	**References**
Demographics	Age, gender, position, work experience				
Practice patterns	2 items	BRIGHT Clinicians' Questionnaire	1) Transplant clinic has a long-term care-coordinator - Care coordinator can be an advanced practice nurse, a transplant coordinator or a social worker, 1 item Response option: yes/no; Dichotomous score<br>2) Having an Advanced Practice Nurse with specialization in alloSCT - 1 item Response option: yes/no; Dichotomous score	No information	(1, 2)
	1 item	BRIGHT Clinicians' Questionnaire	Time spent with the transplant team during follow-up, average time per patient in minutes; Continuous variable	No information	
Level of Chronic Illness Management (CIM)	55 items	BRIGHT Clinicians' Questionnaire ; CIMI-BRIGHT .	1) Self-management Support (19 items)<br>2) Delivery System Design (20 items)	**Content validity**: Scale content validity= 0.86	(1, 2)

		Healthcare worker's perspective of chronic illness management implemented in their follow-up transplant program	3) Clinical Decision Support (10 items)<br>5) Clinical Information System (6 items)<br>5-point Likert scale ranging from '1= strongly disagree' to 4= strongly agree'; (5=don't know; set to missing). Higher scores correspond with higher level of CIM implemented. Total score ranging from 0-4.	**Interrater reliability** pilot tested: ranging between 75% and 85%(11)<br>52 of originally 55 items retained in a unidimensional scale, with a Cronbach's alpha of 0.94	
Preparedness of the Tx team in view of CIM	5 items	BRIGHT clinician questionnaire: Refers to the skills and availability of equipment or tools to facilitate chronic care	4-point Likert scale ranging from '1= strongly disagree' to '4= strongly agree' (5 = don't know; set to missing) with higher scores reflecting higher level of preparedness.	5 of the original 10 items retained in a unidimensional scale, with a Cronbach's alpha of 0.82	
Competencies of the Tx team in view of CIM	24 items	BRIGHT clinician questionnaire: assess core competencies	4-point Likert scale ranging from '1= strongly disagree' to '4= strongly agree' (5 = don't know; set to missing) with higher scores reflecting a higher degree of core competencies.	Unidimensional scale, with a Cronbach's alpha of 0.96	
**3. Transplant Directors' survey**					
Dimension	**Items**	**Variables**	**Instrument/measurement Values/scoring/conceptual definition**	**Psychometrics**	**References**
Stem cell transplant center's structural characteristics	5 items	BRIGHT transplant director questionnaire	1) Type of SCT center- 2 response options: 1) University or 2) regional, community or other hospital; dichotomous variable<br>2) Location of the SCT program - 2 Options: 1) urban, or 2) suburban or rural; dichotomous variable<br>3) Years since start of the SCT program - Time in years between start date of the transplant program and the date of the start of the BRIGHT data collection in a given center; continuous variable<br>4) N of patient who are 1-year post SCT and followed-up - Number of patients regularly being followed up; Continuous variable<br>5) Center size – how many SCT have been performed in the last 5 years- Categories based on total number: small (< 75), medium (75-100), or large (> 100) center	No information	(1, 2)

			6) Multidisciplinarity of the team in follow-up = at least one physician, one nurse and one other discipline, 1 item: describe the members of your team (routinely involved or consult only), Various disciplines listed; check box to be ticked if discipline is part of the team, dichotomous score multidisciplinary (yes/no)		
Practice patterns	8 items	BRIGHT transplant director questionnaire	1) Length of hospital stay after alloSCT- 1 item, average length of stay after alloSCT in days; Continuous variable<br>2) Number of yearly visits scheduled for patients after alloSCT – 3 items number of visits in the first year, between 1 and 2 years; and beyond 3 years<br>3) Formal mental health or psychological evaluation before alloSCT – 1 item, yes/no, dichotomous score<br>4) Formal financial-social evaluation before alloSCT- 1 item yes/no, dichotomous score<br>5) Are patients followed-up by the same clinician when visiting the outpatient clinic – 1 item, 3-point Likert scale ranging from '1= always or nearly always' to '3 rarely or never'; Ordinal variable<br>6) Who is the initial contact in case of after hour questions or emergencies – 1 item, yes/no	No information	(1, 2)
eHealth system in the Transplant Center	4 items	**Added 4 items to Swiss BRIGHT transplant director questionnaire to explore information on application of eHealth system in the Transplant Center**	4 items asking for usage of eHealth applications (yes/no), if yes: specification of application, which members using application, which devices and existing IT team (yes/no)	No information	**(12-15)**
Information on implementation aspects	6 items	**Added 6 items to Swiss BRIGHT transplant director questionnaire to explore Information on implementation aspects** of **an eHealth supported new model of care in the follow-up after HSCT**	6 items asking for perception of implementation aspects on 5-point Likert scale ranging from 1 = yes, absolutely to 5 = no, not at all<br>1) open/willing to implement an eHealth supported new model of care in the follow-up after alloSCT<br>2) Hospital support?	No information	**(12-15)**

			3) Available resources in general to introduce innovations in view of eHealth solutions?<br>4) Sufficient human resources in order to implement the role of an APN?<br>5) sufficient financial resources in order to implement the role of an APN?		

Note. Variables written in bold has been added or adapted for this study based on previous work (1-3, 8-11), theoretical frameworks (14, 15) and evidence (4-7, 12, 13). Abbreviations: BRIGHT = Building research initiative group: chronic illness management and adherence in transplantation; CIM = Chronic Illness Management.

References

1. Berben L, Denhaerynck K, Dobbels F, Engberg S, Vanhaecke J, Crespo-Leiro MG, et al. Building research initiative group: chronic illness management and adherence in transplantation (BRIGHT) study: study protocol. *Journal of advanced nursing* (2015) 71(3):642-54.
2. Denhaerynck K, Berben L, Dobbels F, Russell CL, Crespo-Leiro MG, Poncelet AJ, et al. Multilevel factors are associated with immunosuppressant nonadherence in heart transplant recipients: The international BRIGHT study. *American Journal of Transplantation* (2018).
3. Leppla L, Mielke J, Kunze M, Mauthner O, Teynor A, Valenta S, et al. Clinicians and patients perspectives on follow-up care and eHealth support after allogeneic hematopoietic stem cell transplantation: A mixed-methods contextual analysis as part of the SMILe study. *European Journal of Oncology Nursing* (2020) 45:101723. doi: https://doi.org/10.1016/j.ejon.2020.101723
4. Swiss Confederation. Swiss Health Survey Neuchâtel, Switzerland(2019). Available from: https://www.bfs.admin.ch/bfs/de/home/statistiken/gesundheit/erhebungen/sgb.html
5. Elzi L, Spoerl D, Voggensperger J, Nicca D, Simcock M, Bucher HC, et al. A smoking cessation programme in HIV-infected individuals: a pilot study. *Antivir Ther* (2006) 11(6):787-95. Epub 2007/02/22. PubMed PMID: 17310823.
6. Glanz K, Yaroch AL, Dancel M, Saraiya M, Crane LA, Buller DB, et al. Measures of sun exposure and sun protection practices for behavioral and epidemiologic research. *Arch Dermatol* (2008) 144(2):217-22. Epub 2008/02/20. doi: 10.1001/archdermatol.2007.46. PubMed PMID: 18283179.
7. Gugiu PC, Coryn C, Clark R, Kuehn A. Development and evaluation of the short version of the Patient Assessment of Chronic Illness Care instrument. *Chronic illness* (2009) 5(4):268-76.
8. Vanhoof JM, Vandenberghe B, Geerts D, Philippaerts P, De Mazière P, DeVito Dabbs A, et al. Shedding light on an unknown reality in solid organ transplant patients' self-management: A contextual inquiry study. *Clinical transplantation* (2018):e13314.
9. Vanhoof JM, Vandenberghe B, Geerts D, Philippaerts P, Mazière P, DeVito Dabbs A, et al. Technology Experience of Solid Organ Transplant Patients and Their Overall Willingness to Use Interactive Health Technology. *Journal of Nursing Scholarship* (2017) 50(2):1-12.
10. De Geest S, Deschodt M, INSPIRE Project Team. INSPIRE Project (2019). Available from: https://inspire-bl.unibas.ch/project-description/.
11. Berben L, Russell CL, Engberg S, Dobbels F, De Geest S. Development, content validity and inter-rater reliability testing of the Chronic Illness Management Implementation–Building Research Initiative Group: Chronic Illness Management and Adherence in Transplantation: An instrument to assess the level of chronic illness management implemented in solid organ transplant programmes. *Int J Care Coord* (2014) 17(1-2):59-71.
12. World Health Organization. Strengthening people-centred health systems in the WHO European Region: framework for action on integrated health services delivery Copenhagen, Denmark: Regional Committee for Europe, (2016) [cited 2020 28.07.]. Available from: https://www.euro.who.int/__data/assets/pdf_file/0004/315787/66wd15e_FFA_IHSD_160535.pdf?ua=1.
13. World Health Organization. eHealth at WHO Geneva, Switzerland: eHealth Unit (2020) [cited 2020 March 11,]. Available from: https://www.who.int/ehealth/about/en/.
14. Gee PM, Greenwood DA, Paterniti DA, Ward D, Miller LMS. The eHealth enhanced chronic care model: a theory derivation approach. *Journal of medical Internet research* (2015) 17(4):e86. doi: 10.2196/jmir.4067.
15. Pfadenhauer LM, Gerhardus A, Mozygemba K, Lysdahl KB, Booth A, Hofmann B, et al. Making sense of complexity in context and implementation: the Context and Implementation of Complex Interventions (CICI) framework. *Implementation science* (2017) 12(1):21.
