# Supplementary material for: Context-specific adaptation of an eHealth-facilitated, integrated care model and tailoring its implementation strategies—A mixed-methods study as a part of the SMILe implementation science project
Source: Front Health Serv. 2023 Feb 17;2:977564. doi: 10.3389/frhs.2022.977564 (PMC10012712; doi:10.3389/frhs.2022.977564)
Supplement: Supplementary file 2 [file Table2.pdf]

Supplementary Table 2. Patients' and clinicians' perspectives on practice patterns and self-management support in view of Chronic Illness Management

**Patients' perspective**			
**Dimensions**	**Variables**	**Results (N = 60)**	
**Practice Patterns**	**Placement of follow-up care appointments**		
	▪ Only at transplant center, n, %	35	58%
	▪ Also at external center, n, %	25	42%
	Cantonal Hospital Luzern, n, %	6	10%
	University Hospital Bern, n, %	16	27%
	Other, n, %	3	5%
	**Adherence to appointment keeping**		
	▪ Transplant center: Never missed, n, %	60	100%
	▪ External center: Never missed, n, %	24	96%
	o Missed once, n, %	1	4%
	**Time spent with the transplant team during follow-up: Transplant center**		
	▪ Physicians, n, mean (SD)	60	23 min (±8 min.)
	▪ Nurses, n, mean (SD)	59	14 min. (±9 min.)
	▪ Psycho-oncologist, n, mean (SD)	4	49 min. (±8 min.)
	▪ Nutritionist, n, mean (SD)	3	30 min. (±0 min.)
	▪ Physiotherapist, n, mean (SD)	1	30 min.
	▪ Social worker, n, mean (SD)	1	30 min.
	**Time spent with the transplant team during follow-up: External centers**		
	▪ Physicians, n, mean (SD)	25	24 min. (±11min.)
	▪ Nurses, n, mean (SD)	23	12 min. (±7 min.)
	▪ Psycho-oncologist, n, mean (SD)	3	52 min (±8 min.)
	▪ Nutritionist, n, mean (SD)	0	0
	▪ Physiotherapist, n, mean (SD)	1	45 min.
	▪ Social worker, n, mean (SD)	0	0
**Patient's perspective of CIM implemented in their follow-up care program**	Short version of the Patient Assessment of Chronic Illness Care (PACIC) instrument; mean, SD (range)	30.6	±7.8 (11 – 55)

Patients Self-Report on Self-Management and Health Behaviors	**Level of physical activity**		
	**Moderate physically active,** n, **%**	58	97%
	How long per day are you moderate active (in minutes)?, n, mean (SD)		132 min. (± 130 min.)
	Days per week moderate physical active, mean (SD)	6 days	±2 days
	▪ 1 day, n (%)	0	0
	▪ 2 days, n (%)	3	5%
	▪ 3 days, n (%)	4	7%
	▪ 4 days, n (%)	5	8%
	▪ 5 days, n (%)	8	13%
	▪ 6 days, n (%)	5	8%
	▪ 7 days, n (%)	33	55%
	▪ Currently not possible due to illness	2	3%
	**Vigorous physically active, yes (%)**	44	73%
	How long per day are you **vigorous** active (in minutes)?, mean (SD)		62 min (± 50min.)
	Days per week **vigorous** physical active, mean (SD)	3 days	±2 days
	▪ 1 day, n (%)	6	10%
	▪ 2 days, n (%)	14	24%
	▪ 3 days, n (%)	8	13%
	▪ 4 days, n (%)	0	0%
	▪ 5 days, n (%)	1	2%
	▪ 6 days, n (%)	0	0%
	▪ 7 days, n (%)	3	5%
	▪ Currently not possible due to illness	16	25%
	**Currently smoking**		
	▪ Yes, n (%)	2	3%
	▪ No, stopped <one year, n (%)	6	10%
	▪ No, stopped > one year, n (%)	13	22%
	▪ No, never smoked, n (%)	39	65%
	**Level of alcohol consumption**		
	▪ Yes, n (%)	27	45%
	▪ No, stopped <one year, n (%)	13	22%
	▪ No, stopped > one year, n (%)	11	18%
	▪ No, never have drunken alcohol, n (%)	9	15%
	**If yes: Frequency of alcohol consumption**		

	▪ One to three times/month, n (%)	9	32%
	▪ Several times weekly, n (%)	3	11%
	▪ Once or twice per week, n (%)	15	54%
	▪ Three times a day or more, n (%)	1	4%
	**If yes: Amount of alcohol consumption**		
	▪ Up to 1dl, n (%)	4	14%
	▪ 2 dl, n (%)	18	64%
	▪ 3 to 4 dl, n (%)	5	18%
	**<u>Sun protection measures</u>**		
	**Sun exposure on weekdays (10:00am – 4:00pm) yes, n (%)**	58	97%
	How many hours do you spend outside on a weekday? mean (hours), SD		2 hours (±1 hour)
	▪ 30min or less, n (%)	19	33%
	▪ 31min up to 1hour, n (%)	22	38%
	▪ 2 hours, n (%)	12	21%
	▪ 3 hours, n (%)	2	3%
	▪ 4 hours, n (%)	3	5%
	**Sun exposure on weekends (10:00am – 4:00pm) yes, n (%)**	57	95%
	How many hours do you spend outside on weekends? mean (hours), SD		3 hours (±1,5 hours)
	▪ 30min or less, n (%)	13	23%
	▪ 31min up to 1hour, n (%)	8	14%
	▪ 2 hours, n (%)	15	26%
	▪ 3 hours, n (%)	14	25%
	▪ 4 hours, n (%)	6	11%
	▪ 5 hours, n (%)	1	2%
**Patient's perspective of CIM implemented in their follow-up care program**	**Short version of the Patient Assessment of Chronic Illness Care (PACIC) instrument** mean, SD (range)	30.6	±7.8 (11 – 55)
**EHealth openness**	**Patients' technology openness on Self-Management support**	See supplementary Figure 4a.-4b	
	**Patients' technology openness on Delivery System Design**	See supplementary Figure 4a.-4b	
	**Patients' technology openness on Clinical Information System**	See Table 4	
	Would you agree to input your wellbeing, your vital signs and symptoms?		
	▪ no, I wouldn't agree, n (%)	10	17%
	▪ yes, once a week, n (%)	23	38%

	▪ yes, every third day, n (%)	12	20%
	▪ yes, daily, n (%)	15	25%
	If there was a reminder function, which one would you prefer?		
	▪ no, I don't want a reminder at all, n (%)	13	22%
	▪ no, I don't want a daily reminder, n (%)	21	36%
	▪ yes, but just once a day for all values, n (%)	22	38%
	▪ yes, for every single value, n (%)	2	3%
	If there was a reminder function, which one would you prefer?		
	▪ I don't want a reminder function, n (%)	12	21%
	▪ individually, e.g., chosen picture, n (%)	15	26%
	▪ acoustic sound which has to be turned off, n (%)	4	7%
	▪ written, with pop-up message, n (%)	27	47%
	Would you like to have an updated electronical medication plan?		
	▪ yes	30	50%
	Would you feel confident to input your medication plan and to update it after appointments by yourself?		
	▪ no, this would be too much effort	31	54%
	▪ no, I don't feel confident	1	2%
	▪ yes, but only if checked by professional	6	11%
	▪ yes, I could do by myself	19	33%
	Would you agree to mark your medication intake electronically?		
	▪ no, this would be too much effort	34	58%
	▪ no, I'm not feeling confident	1	12%
	▪ yes, once a day	16	2%
	▪ yes, after every intake	8	14%
	Would you consider using a robot which can support you with the housekeeping?		
	▪ no	35	59%
	▪ I don't know	5	9%
	▪ maybe	11	19%
	▪ yes	8	14%
	Would you consider using a robot to keep you company at home?		
	▪ no	51	85%
	▪ I don't know	0	0%
	▪ maybe	6	10%

	▪ yes	3	5%
**Clinicians' perspective**			
**Dimensions**	**Variables**	**Results (N = 5)**	
**Self-Management Support**		**n**	**Yes, n (%)**
	1. Healthcare workers have enough time to assess, educate and support patients in self-management (e.g., medication taking, smoking, physical activity, sun protection, diet)	5	**2 (40%)**
	2. The transplant team routinely works with patients to identify clear, measurable and workable self-management goals	4	**0 (0%)**
	3. The transplant team helps all patients set up action plans for medication adherence	4	3 (75%)
	4. Before making or changing a treatment plan, the transplant team discusses the alternatives with the patient and asks them about their preferences	5	4 (80%)
	5. The transplant team lets patients decide on the treatment options they prefer	4	**1 (25%)**
	6. The transplant team lets patients decide on self-management goal(s)	4	**1 (25%)**
	7. We always ask patients if they agree with their treatment plans	4	4 (100%)
	8. The transplant team routinely assesses obstacles patients believe prevent them from following their treatment plans	4	2 (50%)
	9. The transplant team routinely assesses each patients' ability to follow the agreed treatment plan	4	**1 (25%)**
	10. The transplant team checks during follow-up if patient is dealing effectively with the treatment plan	4	2 (50%)
	11. The transplant team gives each patient a copy of the agreed treatment plan, including information on self-management	4	**1 (25%)**
	12. The transplant team routinely asks patients to report on their self-management efforts	5	**0 (0%)**
	13. The transplant team routinely uses interpreters for foreign language patients	5	3 (60%)
	14. During follow-up visits, the transplant team routinely reviews, together with the patient, data on self-management	4	**1 (25%)**
	15. The transplant team routinely conducts follow-up contacts within 2 weeks for all patients who have set a new self-management goal	4	**0 (0%)**
	16. The transplant team routinely follows up with the patient within 2 weeks on all patients who have started a new medication regimen or who have set a new self-management goal.	4	0 (0%)

	17. The transplant team routinely assesses patients for their individual concerns or goals	4	4 (100%)
	18. The transplant team routinely refers patients to community services that can help them self-manage their care after leaving the hospital	4	**1 (25%)**
	19. If the transplant team refers patients the team routinely works directly with these agencies to coordinate care for individual patients	4	3 (75%)
	20. If the transplant team refers patients, we routinely follow up with patients to assess their progress	4	2 (50%)
**Delivery System Design**	21. Inpatient and outpatient services are coordinated	5	4 (80%)
	22. Services are coordinated across different healthcare providers	5	**1 (20%)**
	23. Services are coordinated over time	5	**0 (0%)**
	24. There is a specific long-term care coordinator	5	**0 (0%)**
	25. Patients are strongly encouraged to see the same healthcare workers over time	5	2 (40%)
	26. Patients are assessed when they have concerns/questions beside appointments	4	4 (100%)
	27. Outpatient follow-up appointments are scheduled with patients at the end of visit	5	5 (100%)
	28. Patients who cancel their follow-up visits are contacted to reschedule	4	4 (100%)
	29. Patients who cancel their follow-up visit are contacted to identify & solve problems that prevent them from their visits	4	4 (100%)
	30. Healthcare workers are given incentives for effective processes or improved outcomes	4	**1 (25%)**
	31. Patients are given incentives for effective self-management and improved outcomes	5	**1 (20%)**
	32. Senior and other influential leaders clearly help improve the quality of care	5	5 (100%)
	33. The transplant program has a system for routinely monitoring the quality of care	4	4 (100%)
	34. The transplant program has a highly organized quality improvement process	5	4 (80%)
	35. All members of the transplant program's team routinely take part in quality improvement	5	**2 (40%)**
	36. The achievements of quality improvement processes are reported to the public	4	2 (50%)
	37. The transplant program is based on a system of interdisciplinary team care	5	5 (100%)
	38. Team members are given roles and responsibilities for tasks according to their professional strengths and abilities	5	5 (100%)
	39. Clinicians' time is freed by shifting routine jobs to clinical assistants	4	**1 (20%)**
	40. Each member of the healthcare team is respected for his/her unique areas of expertise and contributions to overall patient care	5	5 (100%)

**Clinical Decision Support System**	41. Written guidelines for care are easily available	4	4 (100%)
	42. Written guidelines for care are supported by education/courses	5	5 (100%)
	43. Written guidelines for care are built into care through prompts/reminders	4	2 (50%)
	44. Care guidelines are built into the information system through computerized prompts/reminders	4	**1 (25%)**
	45. The transplant team's information system automatically gives healthcare workers specific guidance for individual patient care (e.g., reminders for visit, blood test)	4	**1 (25%)**
	46. The transplant team's information system automatically flags patients who are overdue for routine follow-up	4	**0 (0%)**
	47. The transplant team's information system is used to give feedback to individual healthcare workers	4	**1 (25%)**
	48. The transplant team's information system is used to give feedback to the transplant team about the quality of care they are delivering and identify areas for improvement	4	**0 (0%)**
	49. The transplant team's information system is used to give information about trends in the transplant population	4	2 (50%)
**Clinical Information Systems**	50. Patients have access to their medical records or personal health records	4	2 (50%)
	51. Electronic medical records are available to all members of the transplant team	5	3 (60%)
	52. Electronic medical records are used by all members of the transplant team	5	**2 (40%)**
	53. The same information about individual patients is accessible in- and outpatient	5	**2 (40%)**
	54. The transplant team uses information systems to monitor patients at home	5	**0 (0%)**
	55. The transplant team uses information systems for research purposes	4	2 (50%)
**Level of CIM**	BRIGHT Clinicians' Questionnaire; CIMI-BRIGHT; mean, SD (range)	2.92	± 0.58 (2.49 – 3.87)
**Preparedness of the Tx team in view of CIM**	BRIGHT clinician questionnaire, mean, SD (range)	3.14	± 0.40 (2.80 – 3.70)
**Competencies of the Tx team in view of CIM**	BRIGHT clinician questionnaire, mean, SD (range)	3.00	± 0.59 (2.42 – 3.67)

Note. Assessment of Chronic Illness Management (CIM) level from patients' and clinicians' perspectives by completing the adapted BRIGHT patient survey (N = 60), respectively the investigator-developed CIMI-BRIGHT clinician questionnaire (N = 5). Values highlighted in bold were agreed by less than 50% of clinicians. Abbreviations. min. = minutes, SD = standard deviation.
