# Supplementary material for: Context-specific adaptation of an eHealth-facilitated, integrated care model and tailoring its implementation strategies—A mixed-methods study as a part of the SMILe implementation science project
Source: Front Health Serv. 2023 Feb 17;2:977564. doi: 10.3389/frhs.2022.977564 (PMC10012712; doi:10.3389/frhs.2022.977564)
Supplement: Supplementary file 3 [file Table3.pdf]

Supplementary Table 3. Ordinal Logistic Regression determining the association between variables and patients' perceptions of the importance to new technologies

Technology	Parameter		OR	Significance	95% CI for Exp(B) Lower bound	Upper bound
New technologies that support physical activity	Age	<60 years	2.57	0.1717	0.67	10.38
		>60 years (Ref)				

Technology	Parameter		OR	Significance	95% CI for Exp(B) Lower bound	Upper bound
New technologies that support physical activity	Gender	Female	1.55	0.4442	0.50	4.82
		Male (Ref)				
	Marital Status	Married / Living with partner	1.37	0.5862	0.44	4.37
		Divorced / Separated	2.43	0.4224	0.27	22.35
		Single (Ref)				
	Education	Professional Education	3.33	0.1308	0.70	16.42
		University-Entrance Diploma	2.64	0.3772	0.32	24.63
		College	14.59*	0.0094	2.03	120.58
		University	3.47	0.2049	0.50	24.22
		Secondary School (Ref)				
	Employment	Retired	0.92	0.9103	0.21	4.10
		Working part time	0.81	0.7811	0.18	3.73
		Working full time	0.18	0.0632	0.03	1.10
		Unable to work (Ref)				
	Months since alloSCT		0.95	0.0709	0.90	1.00

Technology	Parameter		OR	Significance	95% CI for Exp(B) Lower bound	Upper bound
New technologies that measure physical activity	Age	<60 years	3.55	0.0730	0.91	14.79
		>60 years (Ref)				
	Gender	Female	0.87	0.8043	0.28	2.66

		Male (Ref)				
	Marital Status	Married / Living with partner	2.13	0.2001	0.67	6.89
		Divorced / Separated	7.59	0.0796	0.82	86.24
		Single (Ref)				
	Education	Professional Education	0.80	0.7638	0.18	3.50
		University-Entrance Diploma	0.97	0.9782	0.11	9.27
		College	4.40	0.1246	0.68	30.90
		University	0.46	0.3960	0.07	2.80
		Secondary School (Ref)				
	Employment	Retired	5.02*	0.0492	1.05	26.57
		Working part time	3.22	0.1536	0.65	16.49
		Working full time	0.57	0.5170	0.10	3.20
		Unable to work (Ref)				
	Months since alloSCT		0.92	0.0049	0.87	0.97

					95% CI for Exp(B)	
**Technology**	**Parameter**		**OR**	**Significance**	**Lower bound**	**Upper bound**
New technologies that provide information about healthy eating	Age	<60 years	4.41*	0.0369	1.12	18.72
		>60 years (Ref)				
	Gender	Female	1.11	0.8579	0.36	3.46
		Male (Ref)				
	Marital Status	Married / Living with partner	1.11	0.8460	0.37	3.30
		Divorced / Separated	3.15	0.3467	0.29	39.10
		Single (Ref)				
	Education	Professional Education	1.50	0.5921	0.37	6.68
		University-Entrance Diploma	0.23	0.1807	0.03	2.03
		College	3.09	0.2516	0.46	22.24
		University	1.25	0.8065	0.21	7.43
		Secondary School (Ref)				

	Employment	Retired	2.10	0.3308	0.48	9.77
		Working part time	1.22	0.7894	0.28	5.28
		Working full time	0.29	0.1528	0.05	1.58
		Unable to work (Ref)				
	Months since alloSCT		0.98	0.4752	0.93	1.03

					95% CI for Exp(B)	
**Technology**	**Parameter**		**OR**	**Significance**	**Lower bound**	**Upper bound**
New technologies that provide information about infection prevention (e.g., wearing a mask)	Age	<60 years	4.87*	0.0349	1.14	22.18
		>60 years (Ref)				
	Gender	Female	1.21	0.7423	0.39	3.81
		Male (Ref)				
	Marital Status	Married / Living with partner	0.97	0.9577	0.32	2.91
		Divorced / Separated	2.53	0.3702	0.33	20.83
		Single (Ref)				
	Education	Professional Education	2.70	0.1810	0.63	11.94
		University-Entrance Diploma	0.61	0.6474	0.07	5.27
		College	4.24	0.1297	0.67	28.91
		University	3.18	0.1980	0.55	19.31
		Secondary School (Ref)				
	Employment	Retired	4.65	0.0541	0.99	23.10
		Working part time	1.26	0.7503	0.29	5.31
		Working full time	0.81	0.8141	0.14	4.88
		Unable to work (Ref)				
	Months since alloSCT		0.96	0.1510	0.91	1.01

					95% CI for Exp(B)	
**Technology**	**Parameter**		**OR**	**Significance**	**Lower bound**	**Upper bound**
New technologies that provide information about correct hand disinfection	Age	<60 years	5.62*	0.0250	1.27	26.66
		>60 years (Ref)				
	Gender	Female	1.78	0.3247	0.57	5.75

		Male (Ref)				
	Marital Status	Married / Living with partner	1.20	0.7601	0.38	3.79
		Divorced / Separated	2.82	0.3254	0.36	23.75
		Single (Ref)				
	Education	Professional Education	4.01	0.0782	0.86	19.36
		University-Entrance Diploma	0.89	0.9131	0.10	8.03
		College	5.63	0.0806	0.83	41.48
		University	5.02	0.0938	0.78	34.51
		Secondary School (Ref)				
	Employment	Retired	7.40*	0.0154	1.51	39.36
		Working part time	2.52	0.2383	0.54	11.89
		Working full time	1.07	0.9417	0.18	6.78
		Unable to work (Ref)				
	Months since alloSCT		0.96	0.1147	0.91	1.01

					95% CI for Exp(B)	
**Technology**	**Parameter**		**OR**	**Significance**	**Lower bound**	**Upper bound**
New technologies that regularly consume vital signs (e.g. heart rate, blood pressure) and give automatic feedback on whether the values are normal	Age	<60 years	2.66	0.1756	0.66	11.42
		>60 years (Ref)				
	Gender	Female	0.91	0.8725	0.29	2.86
		Male (Ref)				
	Marital Status	Married / Living with parter	0.45	0.1525	0.15	1.33
		Divorced / Separated	1.95	0.5116	0.26	15.37
		Single (Ref)				
	Education	Professional Education	2.85	0.1631	0.66	12.89
		University-Entrance Diploma	0.38	0.3782	0.04	3.16
		College	4.33	0.1206	0.69	28.88
		University	5.27	0.0730	0.87	33.84
		Secondary School (Ref)				
	Employment	Retired	2.28	0.2808	0.51	10.60

		Working part time	3.17	0.1380	0.71	15.28
		Working full time	1.72	0.5222	0.33	9.35
		Unable to work (Ref)				
	Months since alloSCT		0.95	0.0576	0.90	1.00

					95% CI for Exp(B)	
**Technology**	**Parameter**		**OR**	**Significance**	**Lower bound**	**Upper bound**
New technologies that regularly consume symptoms (e.g. sickness, diarrhea) and give automatic feedback on whether the values are normal	Age	<60 years	2.80	0.1531	0.70	12.06
		>60 years (Ref)				
	Gender	Female	0.48	0.2285	0.14	1.60
		Male (Ref)				
	Marital Status	Married / Living with partner	0.37	0.0775	0.12	1.10
		Divorced / Separated	6.85	0.0891	0.78	75.00
		Single (Ref)				
	Education	Professional Education	1.82	0.4283	0.41	8.33
		University-Entrance Diploma	0.22	0.1884	0.02	2.01
		College	2.16	0.4252	0.33	15.04
		University	3.57	0.1663	0.60	22.70
		Secondary School (Ref)				
	Employment	Retired	1.92	0.4003	0.42	9.05
		Working part time	3.03	0.1700	0.65	16.06
		Working full time	1.62	0.5630	0.32	8.55
		Unable to work (Ref)				
	Months since alloSCT		0.94	0.0370	0.89	1.00

					95% CI for Exp(B)	
**Technology**	**Parameter**		**OR**	**Significance**	**Lower bound**	**Upper bound**
New technologies that allow physicians and nurses to monitor vital signs, symptoms, medication intake and physical activity	Age	<60 years	4.87	0.7940	1.14	22.18
		>60 years (Ref)				
	Gender	Female	1.21	0.8963	0.39	3.81
		Male (Ref)				

	Marital Status	Married / Living with partner	0.97	0.3064	0.32	2.91
		Divorced / Separated	2.53	0.3096	0.33	20.83
		Single (Ref)				
	Education	Professional Education	2.70	0.0373	0.63	11.94
		University-Entrance Diploma	0.61	0.4749	0.07	5.27
		College	4.24	0.0601	0.67	28.91
		University	3.18	0.0711	0.55	19.31
		Secondary School (Ref)				
	Employment	Retired	4.65	0.2466	0.99	23.10
		Working part time	1.26	0.2354	0.29	5.31
		Working full time	0.81	0.4603	0.14	4.88
		Unable to work (Ref)				
	Months since alloSCT		0.96	0.0923	0.91	1.01

					95% CI for Exp(B)	
**Technology**	**Parameter**		**OR**	**Significance**	**Lower bound**	**Upper bound**
New technologies that remind patients about their appointments at the transplant center	Age	<60 years	2.16	0.2616	0.56	8.39
		>60 years (Ref)				
	Gender	Female	1.34	0.6093	0.44	4.20
		Male (Ref)				
	Marital Status	Married / Living with partner	0.46	0.1716	0.15	1.38
		Divorced / Separated	1.22	0.8648	0.12	13.91
		Single (Ref)				
	Education	Professional Education	1.80	0.4467	0.40	8.51
		University-Entrance Diploma	0.46	0.4700	0.05	3.86
		College	5.54	0.0771	0.85	39.19
		University	2.18	0.3800	0.38	12.94
		Secondary School (Ref)				
	Employment	Retired	1.20	0.8110	0.27	5.58

	Working part time	3.17	0.1245	0.73	14.55
	Working full time	1.06	0.9454	0.20	5.77
	Unable to work (Ref)				
Months since alloSCT		0.94	0.0222	0.89	0.99

Note. * Statistically significant, $p < .05$
