# Supplementary material for: Context-specific adaptation of an eHealth-facilitated, integrated care model and tailoring its implementation strategies—A mixed-methods study as a part of the SMILe implementation science project
Source: Front Health Serv. 2023 Feb 17;2:977564. doi: 10.3389/frhs.2022.977564 (PMC10012712; doi:10.3389/frhs.2022.977564)
Supplement: Supplementary file 4 [file Datasheet1.pdf]

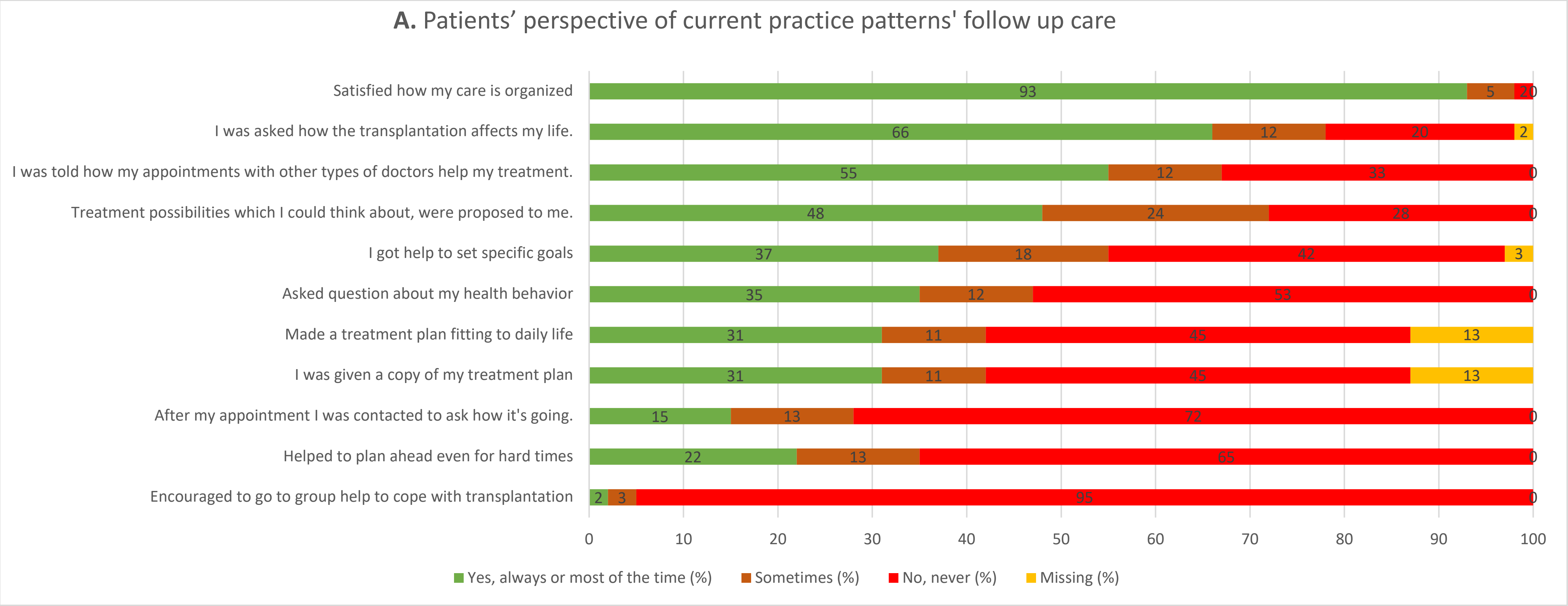
A. Patients’ perspective of current practice patterns' follow up care
Satisfied how my care is organized
I was asked how the transplantation affects my life.
I was told how my appointments with other types of doctors help my treatment.
Treatment possibilities which I could think about, were proposed to me.
I got help to set specific goals
Asked question about my health behavior
Made a treatment plan fitting to daily life
I was given a copy of my treatment plan
After my appointment I was contacted to ask how it's going.
Helped to plan ahead even for hard times
Encouraged to go to group help to cope with transplantation
Yes, always or most of the time (%)
Sometimes (%)
No, never (%)
Missing (%)

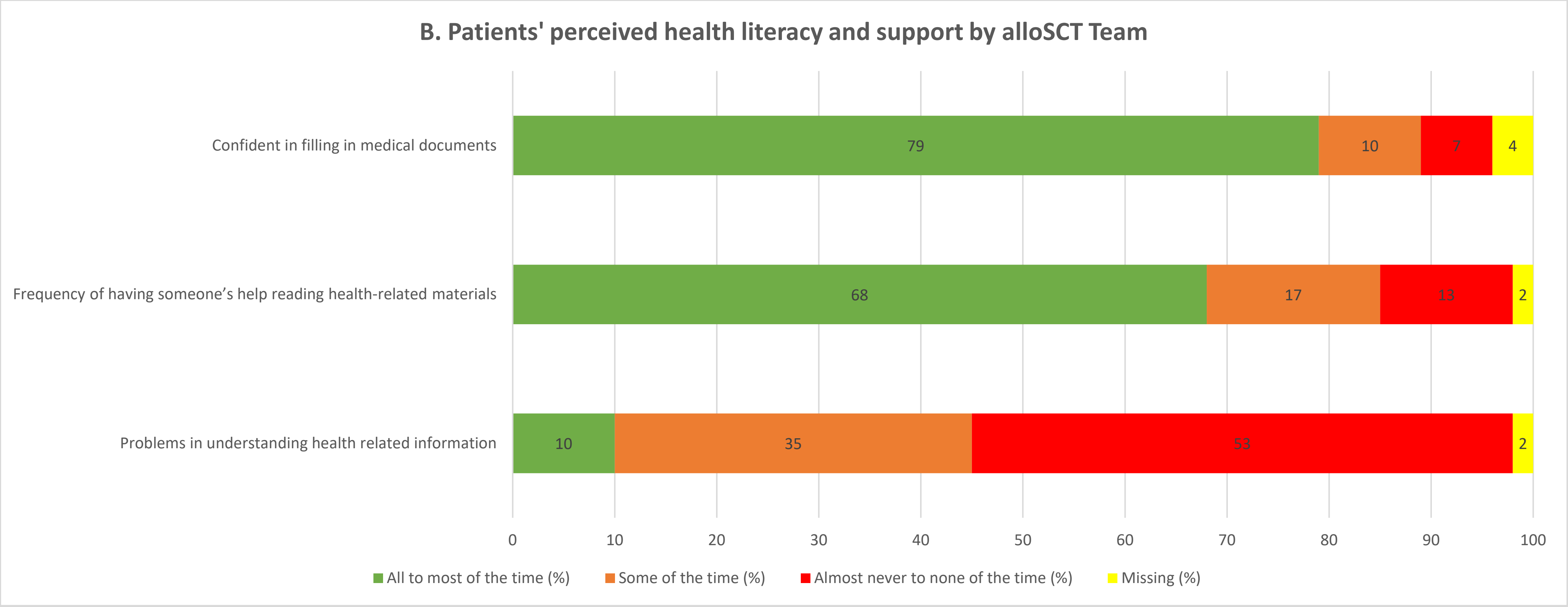
B. Patients' perceived health literacy and support by alloSCT Team
Confident in filling in medical documents
Frequency of having someone's help reading health-related materials
Problems in understanding health related information
79
10
7
4
68
17
13
2
10
35
53
2
0
10
20
30
40
50
60
70
80
90
100
All to most of the time (%)
Some of the time (%)
Almost never to none of the time (%)
Missing (%)

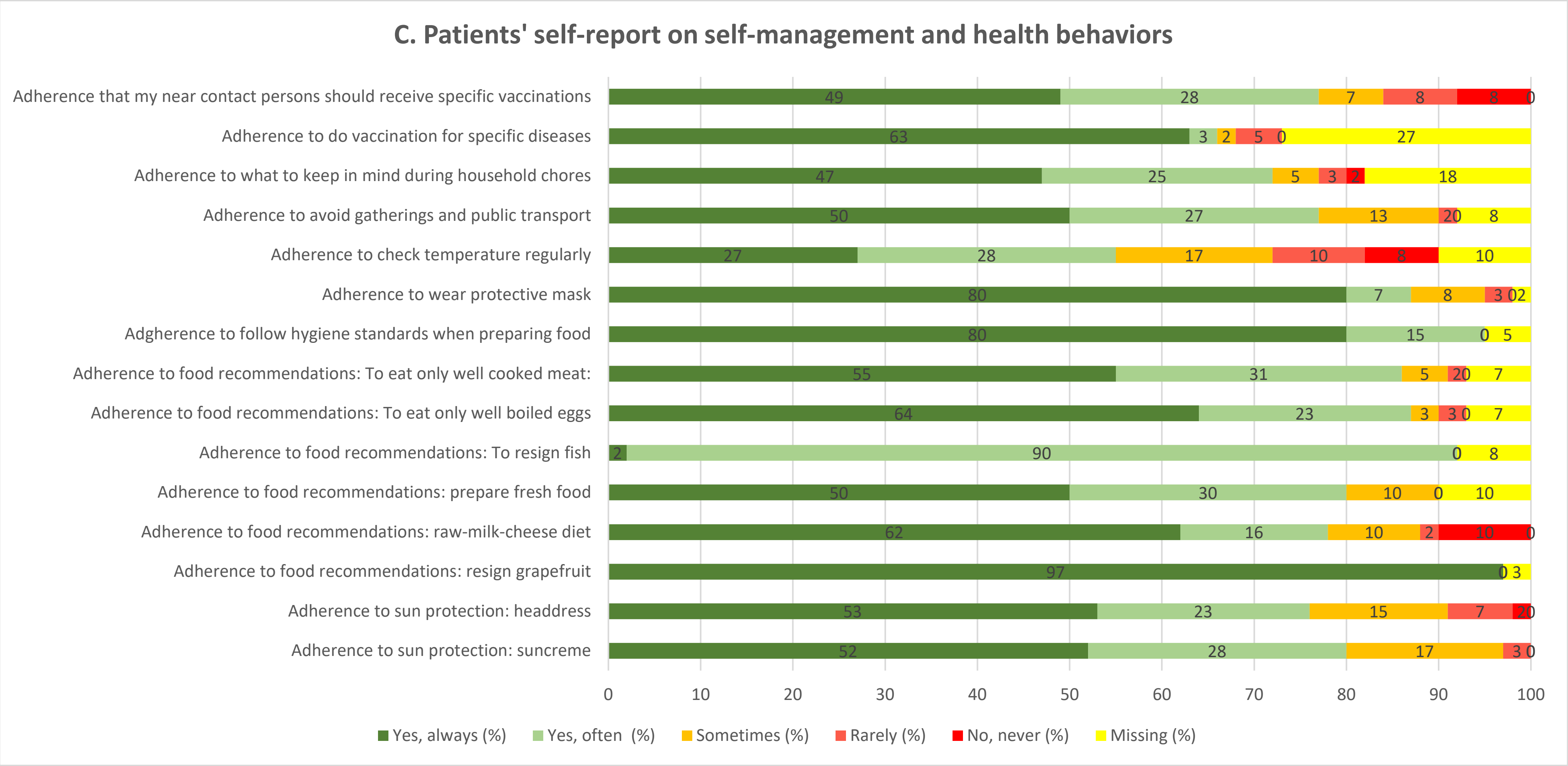

C. Patients' self-report on self-management and health behaviors
Adherence that my near contact persons should receive specific vaccinations
Adherence to do vaccination for specific diseases
Adherence to what to keep in mind during household chores
Adherence to avoid gatherings and public transport
Adherence to check temperature regularly
Adherence to wear protective mask
Adgherence to follow hygiene standards when preparing food
Adherence to food recommendations: To eat only well cooked meat:
Adherence to food recommendations: To eat only well boiled eggs
Adherence to food recommendations: To resign fish
Adherence to food recommendations: prepare fresh food
Adherence to food recommendations: raw-milk-cheese diet
Adherence to food recommendations: resign grapefruit
Adherence to sun protection: headdress
Adherence to sun protection: suncreme
Yes, always (%)
Yes, often (%)
Sometimes (%)
Rarely (%)
No, never (%)
Missing (%)
