# Supplementary material for: Context-specific adaptation of an eHealth-facilitated, integrated care model and tailoring its implementation strategies—A mixed-methods study as a part of the SMILe implementation science project
Source: Front Health Serv. 2023 Feb 17;2:977564. doi: 10.3389/frhs.2022.977564 (PMC10012712; doi:10.3389/frhs.2022.977564)
Supplement: Supplementary file 5 [file Datasheet2.pdf]

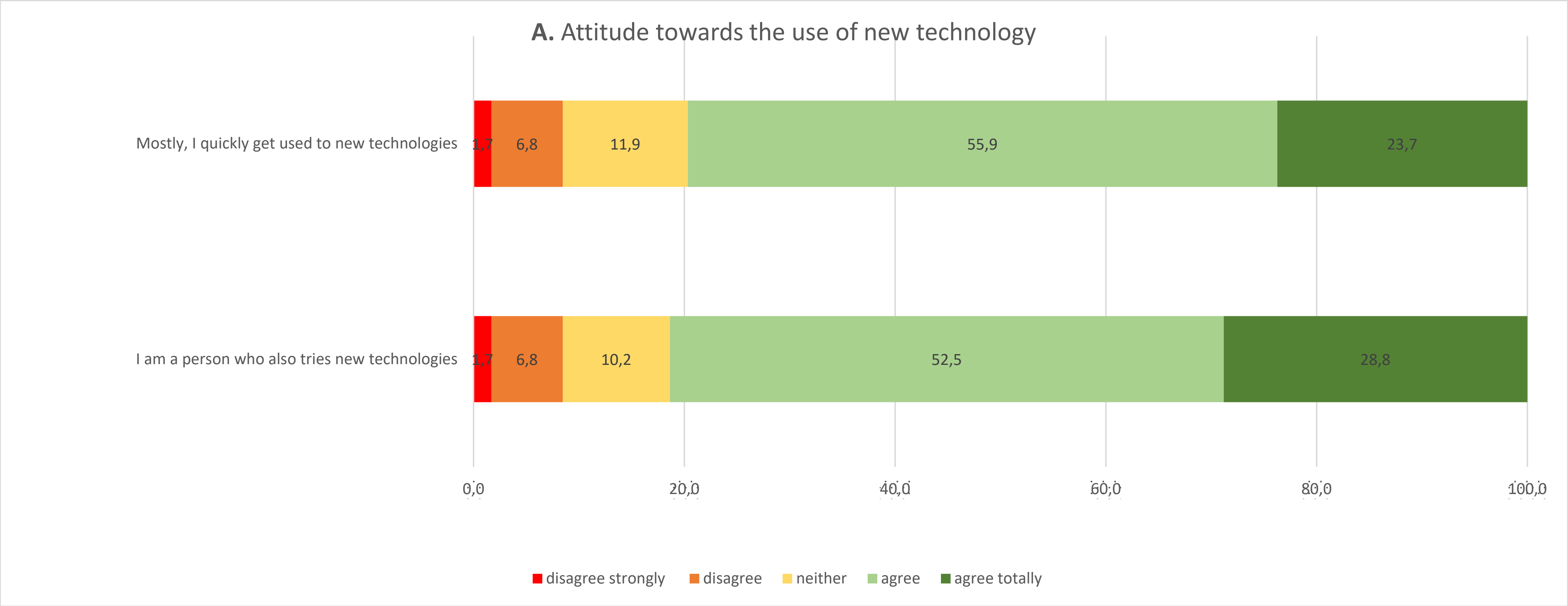
A. Attitude towards the use of new technology
Mostly, I quickly get used to new technologies
1,7
6,8
11,9
55,9
23,7
I am a person who also tries new technologies
1,7
6,8
10,2
52,5
28,8
0,0
20,0
40,0
60,0
80,0
100,0
disagree strongly
disagree
neither
agree
agree totally

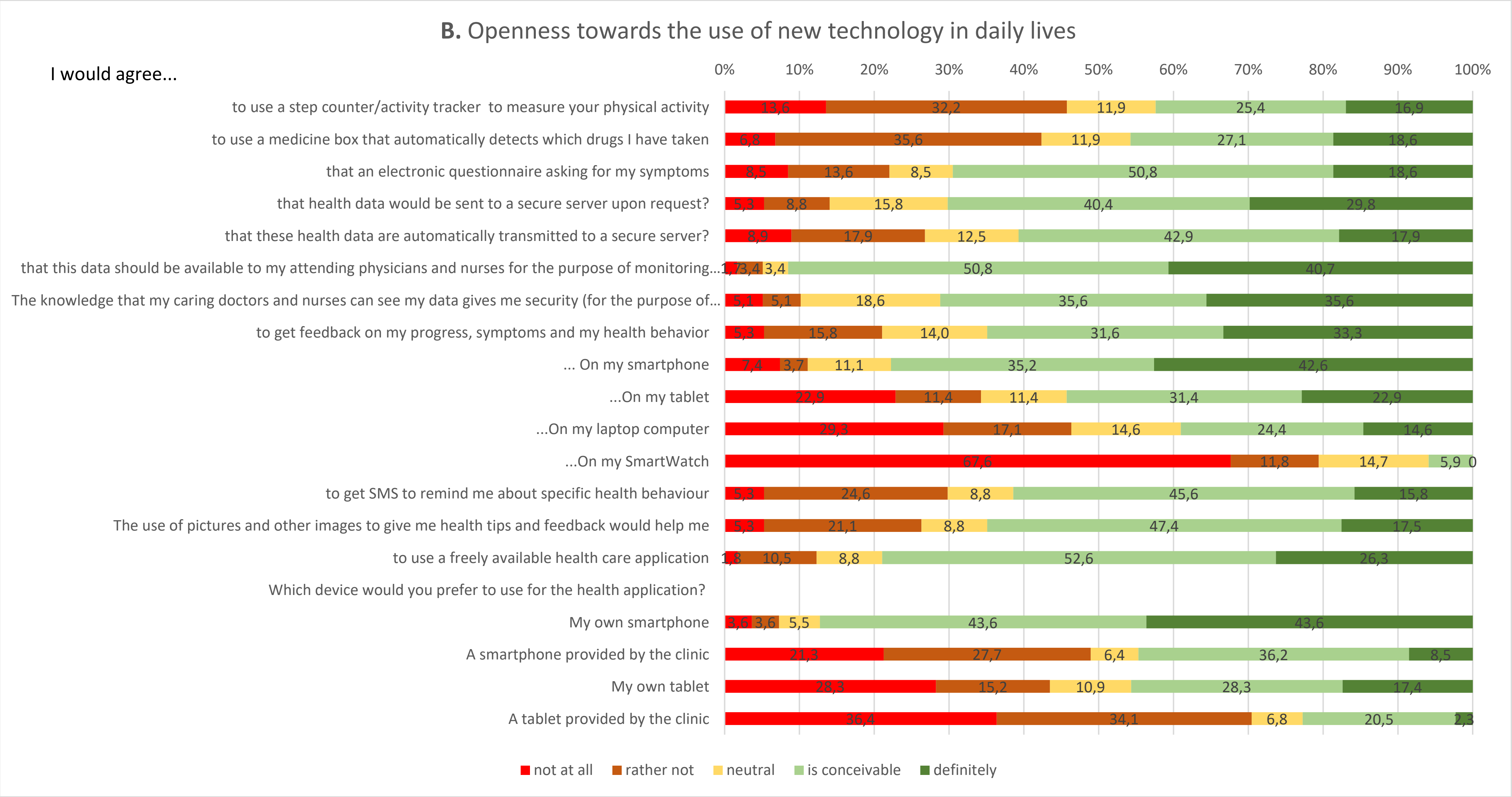
B. Openness towards the use of new technology in daily lives
I would agree...
to use a step counter/activity tracker to measure your physical activity
to use a medicine box that automatically detects which drugs I have taken
that an electronic questionnaire asking for my symptoms
that health data would be sent to a secure server upon request?
that these health data are automatically transmitted to a secure server?
that this data should be available to my attending physicians and nurses for the purpose of monitoring...
The knowledge that my caring doctors and nurses can see my data gives me security (for the purpose of...
to get feedback on my progress, symptoms and my health behavior
... On my smartphone
...On my tablet
...On my laptop computer
...On my SmartWatch
to get SMS to remind me about specific health behaviour
The use of pictures and other images to give me health tips and feedback would help me
to use a freely available health care application
Which device would you prefer to use for the health application?
My own smartphone
A smartphone provided by the clinic
My own tablet
A tablet provided by the clinic
not at all
rather not
neutral
is conceivable
definitely
